# Chitosan-based nanotheranostics integrated dual-modal imaging and combinatorial tumor therapy for EGFR-TKI resistance reversal

**DOI:** 10.1016/j.mtbio.2026.103420

**Published:** 2026-06-29

**Authors:** Fangying Zheng, Yanyun Su, Xianbin Sun, Ding Tan, Ya Wang, Xiumei Li, Yu Gao

**Affiliations:** a Fujian Provincial Key Laboratory of Cancer Metastasis Chemoprevention and Chemotherapy, College of Chemistry, Fuzhou University, Fuzhou, Fujian, 350116, China; b Department of Radiology, The First Affiliated Hospital of Fujian Medical University, Fuzhou, Fujian, 350005, China

**Keywords:** NSCLC, EGFR-TKI resistance, TME remodeling, Combination therapy, Nanotheranostics

## Abstract

Epidermal growth factor receptor tyrosine kinase inhibitors (EGFR-TKIs) are primary treatments for EGFR-mutated non-small cell lung cancer (NSCLC), but acquired resistance limits efficacy. To address this challenge, we developed CsO/IGA, a multifunctional nanoplatform combining osimertinib (AZD9291) with an indocyanine green-gadolinium (ICG-Gd) probe via oleanolic acid (OA)-modified chitosan encapsulation, integrating fluorescence/magnetic resonance imaging (FLI/MRI) with chemo-phototherapy and tumor microenvironment (TME) modulation. CsO/IGA (∼100 nm) demonstrated excellent stability and generated both singlet oxygen and hyperthermia under 808 nm irradiation. The nanoplatform exerted efficient anti-cancer effects to overcome resistance through reactive oxygen species induction, mitochondrial membrane potential reduction, and EGFR/phosphorylated EGFR downregulation in sensitive and resistant NSCLC cells. Notably, the OA component suppressed cancer-associated fibroblasts and α-smooth muscle actin expression, remodeling the TME to enhance tumor penetration. *In vivo* studies confirmed tumor-specific accumulation and deep penetration, FLI/MRI capability, and superior antitumor efficacy through chemo-phototherapy synergy. This work presents a promising OA-integrated theranostic strategy integrating targeted therapy, phototherapy, TME modulation, and diagnostic imaging for overcoming EGFR-TKI resistance in NSCLC.

## Highlights

• ICG-Gd probe achieves dual-modal fluorescence and magnetic resonance imaging.
• CsO/IGA nanoplatform uses oleanolic acid (OA)-modified chitosan to co-deliver ICG-Gd and AZD9291.
• OA-modified chitosan modulates TME by suppressing CAFs for enhanced deep tumor penetration.
• CsO/IGA integrates OA-mediated TME modulation and chemo-phototherapy to reverse EGFR-TKI resistance.
• CsO/IGA combines dual-modal imaging and combinatorial tumor therapy into one system.

## Graphical abstract

**Figure:**
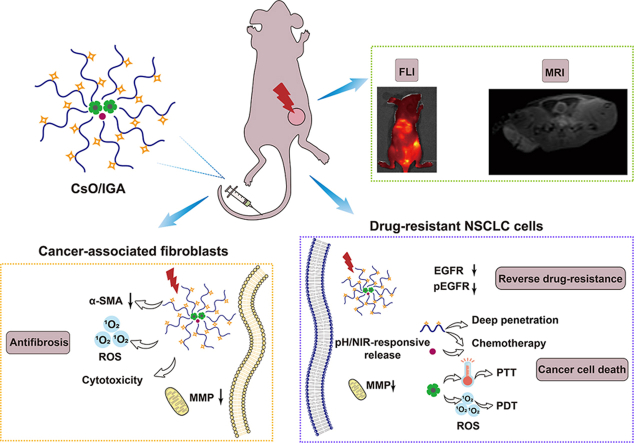

## 1 Introduction

Lung cancer remains the leading cause of cancer-related mortality, with non-small cell lung cancer (NSCLC) accounting for 80% of cases [1,2]. While epidermal growth factor receptor tyrosine kinase inhibitors (EGFR-TKIs) serve as first-line therapy for EGFR-mutant NSCLC [3], acquired resistance inevitably develops through diverse mechanisms including secondary mutations, kinase switches, and tumor microenvironment (TME) alterations [4]. The TME constitutes a complex network comprising cancer-associated fibroblasts (CAFs), abnormal extracellular matrix (ECM) components, and disorganized vasculature, which collectively establish formidable physical and biochemical barriers that limit drug penetration and promote resistance [5]. Emerging evidence suggests that simultaneously targeting malignant cells and modulating the TME may provide an effective strategy to overcome EGFR-TKI resistance [5].

Oleanolic acid (OA) and its derivatives exhibit multifaceted TME-modulating capabilities, including anti-angiogenesis [6], immunomodulation [7], and antifibrotic [8] effects. In our previous studies, OA not only inhibits tumor cell proliferation but also downregulates α-smooth muscle actin (α-SMA)/phosphorylated Smad expression in CAFs while significantly reducing collagen deposition in the TME. These actions collectively mitigate extracellular matrix (ECM) barriers, enhance deep tumor drug penetration, and improve overall antitumor efficacy [9,10]. Therefore, this dual therapeutic strategy combining direct tumor inhibition with TME remodeling through OA-mediated pathways offers a promising approach to overcome EGFR-TKI resistance in NSCLC.

Phototherapy has garnered significant attention as a highly promising anticancer approach due to its remarkable therapeutic efficacy, minimal side effects, non-invasiveness, and precise tumor-targeting capability [11,12]. It includes two main therapeutic strategies: photodynamic therapy (PDT) and photothermal therapy (PTT). In PDT, photosensitizers activated by specific-wavelength light generate cytotoxic reactive oxygen species (ROS) that induce tumor cell death [13]. PTT employs photothermal agents that convert light energy into localized hyperthermia upon irradiation, effectively ablating tumor cells [14]. Notably, the therapeutic effects of phototherapy are independent of genetic mutations, making its combination with EGFR-TKIs particularly advantageous for overcoming mutation-driven resistance and significantly enhancing the overall treatment efficacy.

Nanotheranostics represents an innovative approach that combines diagnostic imaging and therapeutic functions, enabling real-time monitoring of drug distribution, tumor localization, and treatment efficacy assessment [15]. The progress in medical imaging has revolutionized cancer research and clinical practice, particularly in characterizing the complex TME [16]. Among various imaging modalities, magnetic resonance imaging (MRI) offers exceptional advantages including non-invasiveness, high spatial resolution, and absence of ionizing radiation [17], with gadolinium (Gd^3+^) serving as an optimal contrast agent due to its strong paramagnetic properties. Clinically approved linear or macrocyclic Gd^3+^ chelates are routinely used a contrast enhancer in MRI [18]. Complementing MRI, fluorescence imaging (FLI) is widely employed in clinical settings by utilizing near-infrared (NIR)-emitting fluorescent dyes to achieve deeper tissue penetration for intraoperative tumor delineation [19]. Indocyanine green (ICG), an FDA-approved NIR fluorescent dye, has been widely used in clinical diagnostics. Additionally, it exhibits unique phototherapeutic properties, absorbing NIR light to generate both heat and singlet oxygen (^1^O_2_) for combined PTT and PDT [20]. The molecular architecture of ICG featuring sulfonic acid (R-HSO_3_) and Lewis base (-NH) moieties, enables metal chelation capacity [21]. Building on previous work demonstrating ICG-Fe^3+^ complexes for dual-modality imaging and enhanced therapeutic performance [22], we hypothesized that ICG-Gd^3+^ complexation could create a novel dual-modal probe integrating FLI/MRI capabilities with phototherapeutic functions. Chitosan (Cs), a natural cationic polysaccharide, has emerged as an ideal drug carrier due to its excellent biocompatibility, biodegradability, and low toxicity [23]. The abundant amino and hydroxyl groups on Cs allow versatile chemical modifications [24]. In our previous study, OA-modified Cs derivative (CsO) was synthesized, which can self-assemble into nanoparticles with capabilities for passive tumor targeting and enhanced tissue penetration [25]. This CsO-based platform represents an ideal carrier for developing NSCLC nanotheranostics combining imaging and therapeutic agents.

**Scheme 1:**
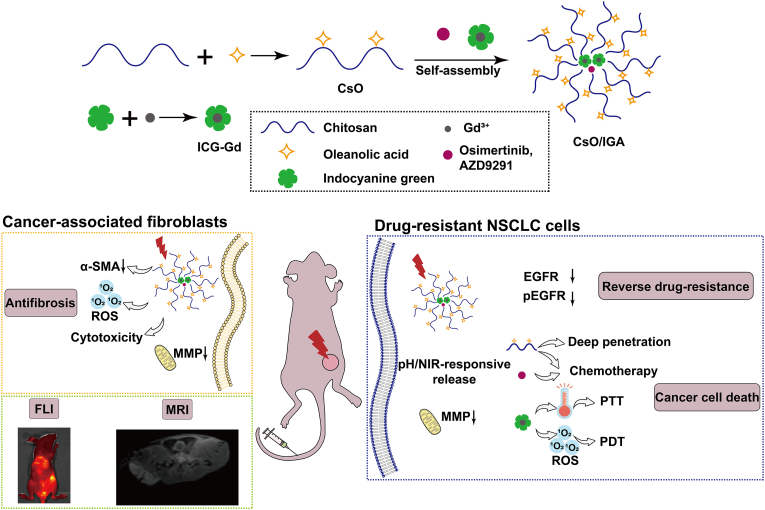
Schematic illustration of the construction and theranostic functions of the CsO/IGA nanotheranostic platform.

Based on the above research background, we propose to develop a nanotheranostic platform for NSCLC treatment by leveraging the metal chelation properties of ICG to create a dual-modal imaging probe (ICG-Gd) through complexation with Gd^3+^. This probe simultaneously enables phototherapy and FLI/MRI. Using our previously developed CsO as a nanocarrier, ICG-Gd and the third-generation EGFR-TKI osimertinib (AZD9291) are co-loaded via self-assembly to form nanoparticles (CsO/IGA). CsO not only can function as an efficient nanocarrier to improve drug delivery, but also exert the antifibrotic effects of OA to remodel the TME by reducing ECM barriers, thereby enhancing deep tumor penetration. The strong fluorescence signal and paramagnetic properties of ICG-Gd enable dual FLI/MRI functionalities. Under laser irradiation, CsO/IGA is designed to synergistically combine PDT/PTT and targeted chemotherapy to overcome EGFR-TKI resistance. CsO/IGA is expected to achieve dual-modal FLI/MRI for precise diagnosis, TME modulation for enhanced drug delivery, and combined photo-chemotherapy for superior antitumor efficacy, ultimately providing a comprehensive solution for reversing EGFR-TKI resistance in NSCLC treatment (Scheme 1).

## 2 Materials and methods

### 2.1 Materials

ICG and 2′,7′-dichlorodihydrofluorescein diacetate (DCFH-DA) was purchased from Bidpharm Co., Ltd. (Shanghai, China). GdCl_3_·6H_2_O was got from Aladdin Biochemical Technology Co., Ltd. (Shanghai, China). Osimertinib (AZD9291) was purchased from Dingguo Changsheng Biotechnology Co., Ltd. (Beijing, China). Fetal novine serum (FBS), Roswell Park Memorial Institute 1640 (RPMI-1640) medium, and Dulbecco's Modified Eagle's medium (DMEM) were got from Meilun Biotechnology Co., Ltd. (Dalian, China). Singlet Oxygen Sensor Green (SOSG) was got from Thermo Fisher Scientific (Shanghai, China). 3-(4,5-Dimethylthiazol-2-yl)-2,5-diphenyltetrazolium bromide (MTT) and Hoechst 33342 were purchased from Tagene Biotechnology Co., Ltd. (Xiamen, China). Rhodamine 123 (Rh 123) were provided by Beyotime Biotechnology Co., Ltd. (Shanghai, China). The primary antibodies of EGFR, phosphorylated EGFR (p-EGFR), glyceraldehyde-3-phosphate dehydrogenase (GAPDH), and horseradish peroxidase (HRP)-conjugated secondary antibodies were purchased from Wanlei bio Co., Ltd. (Shenyang, China). The AKT antibody was purchased from Abmart Pharmaceutical Technology Co., Ltd. (Shanghai, China). Bovine serum albumin (BSA) was purchased from Yancheng Saibao Biotechnology Co., Ltd (Jiangsu, China). Anti-α-smooth muscle actin (α-SMA) fluorescein isothiocyanate (FITC)-conjugated antibody (F3777) was got from Sigma-Aldrich (USA). Transforming growth factor-β1 (TGF-β1) was provided by Novoprotein Scientific Inc. (Suzhou, China). Hyaluronic acid methacryloyl (HAMA) and lithium phenyl-2,4,6-trimethylbenzoyl-phosphinate (LAP) were provide by Yongqinquan Intelligent Equipment Co., Ltd. (Suzhou, China). Alanine aminotransferase (ALT) and aspartate aminotransferase (AST) assay kits were purchased from Jiancheng Bioengineering Research Institute (Nanjing, China). Creatinine (CRE) and urea nitrogen (BUN) assay kits were obtained from Boxbio Co., Ltd. (Beijing, China).

### 2.2 Preparation and characterization of ICG/Gd^3+^ coordinated complex (ICG-Gd)

An aqueous solution of ICG and an aqueous solution of GdCl_3_·6H_2_O were mixed in deionized water (ddH_2_O) at molar ratios ranging from 1:1 to 7:1 (ICG:Gd^3+^). The mixture was stirred at room temperature for 24 h and lyophilized to obtain ICG-Gd complexes at varying ratios. The fluorescence spectra (λ_ex_ = 750 nm) of both ICG and ICG:Gd solutions were then measured.

The ICG-Gd complex at a 5:1 molar ratio was further dialyzed against ddH_2_O using a dialysis bag (MWCO 3500 Da) for 24 h to remove unbound Gd^3+^, and the complexation efficiency was quantified using inductively coupled plasma optical emission spectrometry (ICP-OES).

Aqueous solutions of ICG, ICG-Gd (5:1 molar ratio; ICG concentrations: 0, 10, 20, 40, 80, 160, 320 μM), and gadolinium-1,4,7,10-tetraazacyclododecane-1,4,7,10-tetraacetic acid (Gd-DOTA) solution (Gd^3+^ concentrations: 0, 2, 4, 8, 16, 32, 64 μM) were prepared (1 mL each) and subjected to *T*_*1*_-weighted MRI on a 3.0T MRI scanner (Siemens, German). The scanning parameters included a repetition time (TR) of 650.0 ms, echo time (TE) of 10.0 ms, slice thickness/spacing of 1.0 mm, and a field of view (FoV) of 80 × 80 mm^2^.

### 2.3 Preparation and characterization of self-assembled nanocomplexes

CsO was synthesized by click chemistry and CsO nanoparticles (CsO NPs) were prepared by self-assembly following our previously reported method [25]. Briefly, a CsO solution in DMSO (5 mg/mL) was added dropwise into vigorously stirred ddH_2_O. After stirring for 4 h, the mixture was transferred into a dialysis bag (MWCO: 3500 Da) and dialyzed against ddH_2_O for 24 h to obtain CsO NPs. For drug loading, AZD9291 and ICG-Gd were separately dissolved in DMSO, and added dropwise to the aqueous dispersion of CsO NPs at various weight ratios. The mixtures were stirred at room temperature for 4 h, then dialyzed (MWCO: 3500 Da) in ddH_2_O for 24 h to remove unencapsulated drugs, yielding AZD9291-loaded CsO/A nanocomplexes and ICG-Gd-loaded CsO/IG nanocomplexes, respectively. For the preparation of dual-drug-loaded nanocomplexes, CsO/A at a weight ratio of 200:2 (CsO:AZD9291) were first prepared, followed by the addition of ICG-Gd at different weight ratios. After identical stirring and dialysis procedures, these dual-loaded nanoparticles were designated as CsO/IGA.

The particle size, polydispersity index (PDI), and zeta potential of the nanocomplexes were measured by dynamic light scattering (DLS) on a Malvern Instruments Zetasizer HS III (Malvern, UK) at room temperature. Drug loading was quantified using ultraviolet–visible (UV–Vis) spectroscopy, with standard curves generated from the absorbance of ICG (780 nm) and AZD9291 (317 nm) (Fig. S1). Encapsulation efficiency (EE) was calculated using the following formula:

(1) $$\begin{array}{c} \text{EE}\left(\%\right)=\text{Weight}\;\text{of}\;\text{drug}\;\text{in}\;\text{nanoparticles}/\text{weight}\;\text{of}\;\text{drug}\;\text{inputs}\;\text{totally}\times 100\% \end{array}$$

Considering the limited drug-loading capacity of CsO NPs, optimal formulations were selected based on particle size, PDI, zeta potential, and EE. Unless otherwise specified, the following ratios were selected for subsequent studies: CsO/A (CsO:AZD9291 = 200:2), CsO/IG (CsO:ICG = 200:40), and CsO/IGA (CsO:ICG:AZD9291 = 200:40:2).

The morphology of CsO/IGA was examined using transmission electron microscope (TEM). To assess stability, CsO/IGA was dispersed in H_2_O, phosphate-buffered saline (PBS), RPMI-1640, and RPMI-1640 supplemented with 10% FBS, and the particle size, PDI, and zeta potential were monitored daily for 7 days.

### 2.4 Dual-modality FLI/MRI of CsO/IGA

For FLI analysis, the fluorescence intensity of ICG, ICG-Gd, CsO/IG, and CsO/IGA samples (containing equivalent ICG concentrations) in centrifuge tubes was quantified using a PerkinElmer Caliper IVIS Lumina XR III imaging system (IVIS system, USA).

For MRI evaluation, Gd-DOTA, ICG-Gd, CsO/IG, and CsO/IGA samples (all containing identical Gd^3+^ concentrations) in centrifuge tubes were subjected to transverse *T*_*1*_-weighted MRI under identical scanning parameters as described in Section 2.2.

### 2.5 *In vitro* drug release of CsO/IGA

*In vitro* drug release studies were conducted using PBS containing 20% ethanol at pH 5.5 or 7.4 as release media to simulate acidic lysosomal and physiological conditions, respectively. The release properties of ICG and AZD9291 from CsO/IGA were investigated under different pH conditions with or without laser irradiation. Specifically, CsO/IGA dispersed in PBS in dialysis bags (MWCO: 8000-14000 Da), which were then immersed in 20 mL of release medium. Released samples (1 mL) were collected at predetermined time intervals (1, 2, 4, 6, 8, 12, 24, and 36 h) for absorbance measurement, with an equal volume of fresh medium immediately replenished after each sampling. For the laser irradiation groups, the dialysis bags were exposed to 808 nm laser (2 W/cm^2^) for 3 min at the 2 h and 4 h time points.

### 2.6 Singlet oxygen (^1^O_2_) detection and photothermal properties of CsO/IGA

For ^1^O_2_ detection, aqueous dispersions of ICG-Gd, CsO/IG, and CsO/IGA were mixed with SOSG (5 μM), and exposed to 808 nm laser irradiation (2 W/cm^2^, 3 min). Non-irradiated CsO/IGA and ddH_2_O were used as negative controls. The SOSG fluorescence signal was measured using a microplate reader (Tecan Infinite M200PRO, Switzerland) with excitation/emission of 488/525 nm.

Photothermal performance was evaluated by monitoring temperature changes during 808 nm laser irradiation using an infrared thermal camera (E50, Arlington, VA). The temperature changes of ddH_2_O, CsO, ICG, ICG-Gd, CsO/IG, and CsO/IGA (with equivalent 15 μg/mL ICG) under 808 nm laser irradiation (1.5 W/cm^2^) for 5 min, the temperature changes of CsO/IGA under laser irradiation at different power densities (1, 1.5, 2, and 2.5 W/cm^2^), and the temperature changes of CsO/IGA containing varying ICG concentrations (5, 10, 15, and 20 μg/mL) under laser irradiation (1.5 W/cm^2^) for 5 min were recorded.

Photothermal stability was assessed through three ON/OFF cycles (5 min irradiation at 1.5 W/cm^2^ followed by 7 min cooling) for ICG, ICG-Gd, and CsO/IGA. The photothermal conversion efficiency (PCE, η) was calculated according to previously established methods [26].

### 2.7 Cell culture and animals

The NSCLC cell lines A549, H1975, and PC9, and mouse embryonic fibroblast cell line NIH3T3 (abbreviated as 3T3), were purchased from the Cell Resource Center of Shanghai Institute for Biological Sciences (Chinese Academy of Sciences, Shanghai, China). TGF-β1-activated NIH3T3 cells (3T3^+^ cells) were obtained by treating 3T3 cells with 10 ng/mL TGF-β1 for 48 h [8,25]. A549, H1975, and PC9 cells were grown in RPMI-1640 medium supplemented with 10% FBS. 3T3 cells were cultured with DMEM supplemented with 10% FBS.

Balb/c female nude mice (18-22 g weight, 4-6 weeks old) were purchased from Fuzhou Wushi Animal Center and housed in specific pathogen-free (SPF) conditions with access to food and water *ad libitum*. All animal procedures were conducted in compliance with the Institutional Animal Care and Use Committee guidelines of Fuzhou University (protocol number: 2024-SG-017).

### 2.8 Development of AZD9291-resistant NSCLC cell lines

To establish NSCLC cell lines with progressive AZD9291 resistance, PC9 cells were induced with gradually increasing concentrations of AZD9291. The cells were cultured in RPMI-1640 medium containing incrementally increasing concentrations of AZD9291 (20-600 ng/mL) with intermittent passaging. Briefly, parental PC9 cells were first exposed to 20 ng/mL AZD9291 for 24 h, followed by four passages to establish the first-generation resistant subline (P1). Subsequent resistant sublines (P2-P6) were generated by repeating this process at progressively higher drug concentrations (50, 80, 150, 300, and 600 ng/mL), with four passages performed at each concentration level. To verify the successful establishment of cell lines with different resistance levels, MTT assays were used to quantify AZD9291 sensitivity across the P1-P6 sublines, and flow cytometry analysis was employed to evaluate CsO/IGA cellular uptake efficiency.

### 2.9 Cellular uptake

A549, H1975, PC9, 3T3^+^, and AZD9291-resistant P5 cells were seeded in 12-well plates and cultured overnight. Cells were then incubated with ICG, ICG-Gd, CsO/IG, and CsO/IGA (containing equivalent 5 μg/mL ICG) at 37 °C for 4 h. Following incubation, cells were washed three times with PBS, trypsinized, and resuspended in PBS for fluorescence intensity measurement using a flow cytometry (Becton Dickinson FACSAria III cell sorter).

For visualization of cellular uptake, A549, H1975, PC9, and P5 cells were seeded into 12-well plates with coverslips on the bottom of each well and cultured overnight. After incubating with ICG, ICG-Gd, CsO/IG, and CsO/IGA (containing equivalent 5 μg/mL ICG) at 37 °C for 4 h, the cells were washed three times with saline, followed by nuclear staining with Hoechst 33342 (10 μg/mL, 15 min) and fixation with 4% paraformaldehyde (15 min). The fluorescence was visualized using confocal laser scanning microscopy (CLSM, Leica TCS SP8, Germany).

### 2.10 MTT assay

To investigate the synergistic photothermal-chemotherapeutic effects of CsO/IGA, the optimal irradiation parameters were firstly screened. PC9 cells seeded in 96-well plates were incubated with varying concentrations of CsO/IGA for 4 h, followed by exposure to 808 nm laser irradiation at different power densities (0.5, 1, or 2 W/cm^2^) for either 1 or 2 min, with parallel non-irradiated controls. After a total incubation period of 24 h, cell viability was determined using the MTT assay according to established protocols [27].

The MTT assay was employed to systematically assess the antiproliferative effects of Cs and CsO on A549, H1975, PC9, P5, and 3T3^+^ cells, the comparative cytotoxicity between free AZD9291 and its nanoformulated counterpart CsO/A in NSCLC cells, and the photothermal-enhanced cytotoxicity of ICG-Gd, CsO/IG, and CsO/IGA with or without laser irradiation (1 W/cm^2^, 1 min).

### 2.11 Measurement of intracellular ROS and mitochondrial membrane potential (MMP)

Intracellular ROS levels were detected using the DCFH-DA probe. A549, H1975, PC9, P5, and 3T3^+^ cells were seeded in 12-well plates and cultured overnight. After incubation with ICG, ICG-Gd, CsO/IG, and CsO/IGA (containing equivalent 5 μg/mL ICG) for 4 h, the laser-treated groups were exposed to 808 nm laser irradiation (1 W/cm^2^, 1 min). The cells were then stained with DCFH-DA for 20 min, after which mean fluorescence intensity was quantified by flow cytometry and visualized using CLSM.

MMP was assessed using Rh 123 probe. PC9 and P5 cells were divided into Control, AZD9291, CsO/IGA, ICG-Gd+L, CsO/IG+L, and CsO/IGA+L treatment groups, while 3T3^+^ cells were divided into Control, CsO, CsO/IGA, ICG-Gd+L, CsO/IG+L, and CsO/IGA+L groups (where “L” indicates laser irradiation; all treatments contained 10 μg/mL ICG with total incubation time of 24 h). After 4 h of incubation, laser-treated groups received 808 nm irradiation (1 W/cm^2^, 1 min). All cells were then stained with Rh 123 for 20 min, followed by fluorescence detection using flow cytometry and microscopic observation via CLSM.

### 2.12 Live/dead cell staining assay

PC9 and P5 cells were incubated for 24 h under the following treatment: Control, CsO, AZD9291, CsO/IGA, ICG-Gd+L, CsO/IG+L, and CsO/IGA+L (all containing 10 μg/mL ICG). The laser-treated groups (“+L″) were irradiated following the same protocol as described in the MMP measurement. After treatment, cells were co-stained with calcein-acetoxymethyl ester (calcein-AM) for live cells and propidium iodide (PI) for dead cells, followed by observation via CLSM.

### 2.13 Western blot analysis

P5 cells were seeded in 6-well plate and treated with AZD9291, CsO/IGA, ICG-Gd+L, CsO/IG+L, and CsO/IGA+L (all containing 5 μg/mL ICG). The laser-treated groups (“+L″) were irradiated following the same protocol as described in the MMP measurement. After 24 h of culture, cells were harvested for protein extraction. The proteins were separated by sodium dodecyl sulfate polyacrylamide gel electrophoresis (SDS-PAGE) until optimal band resolution was achieved, then transferred to polyvinylidene difluoride (PVDF) membranes. Membranes was blocked with 5% BSA (for EGFR/pEGFR and AKT detection) or 5% skim milk (for GAPDH) for 2 h, probed with primary antibodies against EGFR, AKT, or GAPDH at 4 °C for 48 h, and then incubated with an HRP-conjugated secondary antibody for 2 h at room temperature. Chemiluminescent signals were measured using the ChemiDoc XRS system (Bio-Rad, USA) and quantified using ImageJ software. For detection of pEGFR, membranes were stripped with stripping buffer (15 min, room temperature) after EGFR detection, re-blocked, and reprobed with pEGFR antibody following the same protocol.

### 2.14 Immunofluorescence staining

For the immunofluorescence analysis, 3T3 and 3T3^+^ cells were seeded in 24-well plates with coverslips on the bottom. 3T3^+^ cells were individually incubated with Cs, CsO, ICG-Gd, CsO/IG, CsO/IGA, ICG-Gd+L, CsO/IG+L, and CsO/IGA+L for 24 h, with untreated cells serving as the control group. The laser-treated groups (“+L″) were irradiated following the same protocol as described in the MMP measurement. Immunofluorescence staining of treated cells and tumor tissue sections was performed according to established methods [25], and the intracellular fluorescence was observed by CLSM.

### 2.15 Hemolysis assay

Red blood cell suspensions (2% in PBS, 500 μL) were mixed with CsO/IGA dispersions (in PBS, 500 μL) to achieve final ICG concentrations of 1, 2, 10, 15, and 20 μg/mL, respectively. Using ddH_2_O as the positive control and PBS as the negative control, the mixtures were incubated at 37 °C for 3 h. The hemolysis rate was calculated by measuring supernatant absorbance (Abs) using the formula:

(2) $$\begin{array}{c} \text{Hemolysis}\;\text{rate}\;\left(\%\right)=\frac{{\text{Abs}}_{s\text{ample}}-{\text{Abs}}_{\text{negative}}}{{A\text{bs}}_{\text{positive}}-{\text{Abs}}_{\text{negative}}}\times 100\% \end{array}$$

### 2.16 *In vivo* imaging

P5 cells suspended in 100 μL PBS were injected subcutaneously into the right flank of Balb/c nude mice to establish a P5 tumor-bearing mice model. When the tumors reached a volume of approximately 100 mm^3^, mice were randomly divided into groups and injected intravenously with PBS, ICG, ICG-Gd, CsO/IG, and CsO/IGA (1 mg/kg ICG equivalent). *In vivo* FLI was performed at 1, 2, 4, 8, 12, and 24 h post-injection using an IVIS system. For *ex vivo* analysis, mice were sacrificed at 2 h, and the tumors and major organs (heart, liver, spleen, lung, and kidney) were collected for imaging. MRI studies were conducted before and after intravenous injection of Gd-DOTA, ICG-Gd, CsO/IG and CsO/IGA, using a *T*_*1*_-weighted MRI with the scanning parameters including TR of 618.0 ms, TE of 13.0 ms, slice thickness/spacing of 1.0 mm, and FoV of 80 × 80 mm^2^.

### 2.17 Penetration in three dimensional (3D) tumor spheroids and solid tumor

For 3D tumor spheroid studies, PC9 or P5 cells were suspended in 1% (w/v) HAMA solution dissolved in LAP and crosslinked via photopolymerization. The encapsulated cells within the hydrogel were cultured in RPMI-1640 medium for 14 days to form uniform 3D tumor spheroids. Spheroids of comparable size were then incubated with ICG, CsO/IG, and CsO/IGA (each containing 5 μg/mL ICG) for 4 h. Following PBS washing, spheroid penetration was assessed using CLSM.

For solid tumor penetration studies, P5 tumor-bearing mice were intravenously injected with ICG, ICG-Gd, CsO/IG, and CsO/IGA via the tail vein. After 2 h of administration, the mice were euthanized and tumors were harvested. The tumor tissues were sectioned using a freezing microtome (Leica CM 1950, Germany), stained with Hoechst 33342 for 15 min, and imaged by CLSM.

### 2.18 *In vivo* antitumor efficacy

P5 tumor-bearing mice were randomized into five treatment groups when tumor volumes reached approximately 100 mm^3^: PBS control, CsO/IGA, ICG-Gd+L, CsO/IG+L, and CsO/IGA+L (where “L” indicates laser irradiation). Treatment groups received intravenous injections of PBS, ICG-Gd, CsO/IG or CsO/IGA (1 mg/kg ICG equivalent in 100 μL PBS) through the tail vein every two days. For laser-treated groups, tumors were irradiated with an 808 nm laser (1.0 W/cm^2^, 5 min) after 2 h of injection. The weight and tumor volume of mice were measured every two days. Tumor volume (V) was calculated using the formula:

(3) $$\begin{array}{c} V=\text{tumor}\;\text{length}\times {\text{tumor}\;\text{width}}^{2}/2 \end{array}$$

At study endpoints, whole blood was collected from mice in each group, and serum was obtained by centrifugation. The concentrations of AST, ALT, CRE, and BUN were quantified using commercial enzyme-linked immunosorbent assay (ELISA) kits according to the manufacturer's instructions to evaluate the effects of the drug on hepatic and renal function in mice. Additionally, tumors were harvested for hematoxylin and eosin (H&E) staining to evaluate histopathological changes. The heart, liver, spleen, lung, and kidney from mice in each group were homogenized, and the residual Gd^3+^ was detected by ICP-OES.

### 2.19 Statistical analysis

All data are presented as mean ± standard deviation (SD) with at least three replicates. Statistical analysis was performed using one-way analysis of variance (ANOVA) test. ∗p < 0.05, ∗∗p < 0.01, ∗∗∗p < 0.001, and ∗∗∗∗p < 0.0001 were deemed to be significant differences.

## 3 Results and discussion

### 3.1 Dual-modal FLI/MRI ability of ICG-Gd

**Fig. 1:**
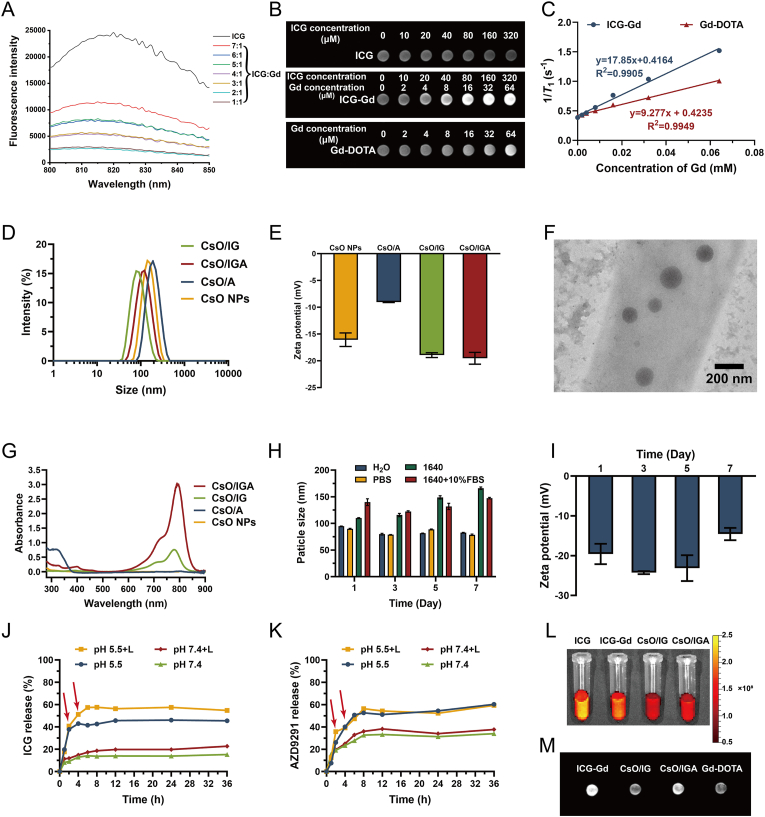
Preparation and characterization of CsO/IGA. (A) Fluorescence spectra of ICG-Gd coordination complexes with different ICG:Gd molar ratios. (B) *T*_*1*_-weighted images of ICG, ICG-Gd, and Gd-DOTA. (C) *T*_*1*_ relaxivity (*r*_*1*_) of ICG-Gd and Gd-DOTA derived from the linear correlation between the *T*_*1*_ relaxation rate (1/*T*_*1*_) and Gd concentration. (D-E) Particle size distribution and zeta potential of CsO NPs, CsO/A, CsO/IG, and CsO/IGA. (F) TEM images of CsO/IGA. (G) UV–Vis absorption spectra of CsO NPs, CsO/A, CsO/IG, and CsO/IGA. (H) Changes in the particle size of CsO/IGA in different media over 1 week. (I) Changes in the zeta potential of CsO/IGA in ddH_2_O over 1 week. (J-K) Cumulative release profiles of ICG and AZD9291 from CsO/IGA in 20% ethanol-PBS at different pH values with or without laser irradiation over 36 h. The red arrow indicates the time point of laser irradiation. (L-M) *In vitro* FLI and MRI of ICG-Gd-based formulations. (For interpretation of the references to colour in this figure legend, the reader is referred to the Web version of this article.)

Previous studies have demonstrated that ICG-Fe^3+^ complexation enables stable dual-modality imaging and enhanced therapeutic performance [22]. Based on the metal complexation capabilities of ICG [21], the ICG-Gd coordinated complexes with dual-modal FLI/MRI capabilities were prepared via electrostatic interactions. A series of complexes with different ICG:Gd molar ratios were prepared, and their fluorescence spectra were measured (Fig. 1A). The complexation of Gd^3+^ caused different degrees of fluorescence quenching in ICG. To ensure sufficient fluorescence signal for FLI while maintaining effective MRI functionality, the Gd^3+^proportion was optimized, and ICG-Gd complex with a 5:1 molar ratio (ICG:Gd) was selected for further study.

Subsequently, the MRI performance of ICG-Gd was evaluated *in vitro* (Fig. 1B and C). While ICG itself lacked MRI signal-enhancing capability, the incorporation of Gd^3+^ significantly improved *T*_*1*_-weighted imaging contrast, which increased with higher complex concentrations. At equivalent Gd^3+^ concentrations, ICG-Gd exhibited substantially stronger contrast enhancement than Gd-DOTA. Linear fitting of the longitudinal relaxation rate (1/*T*_*1*_) as a function of Gd concentrations yielded a *T*_*1*_ relaxivity (*r*_*1*_) of 17.85 mM^−1^ s^−1^ for ICG-Gd, which is nearly twice that of Gd-DOTA (9.28 mM^−1^ s^−1^). These results confirm the superior MRI performance of ICG-Gd.

To assess Gd^3+^ stability and rule out potential dissociation, we quantified the complexation efficiency of Gd^3+^ in ICG-Gd using ICP-OES, which yielded a high value of 89.2%. This robust complexation efficiency indicates minimal free Gd^3+^ in the formulation, ensuring consistent MRI performance and biosafety.

### 3.2 Preparation and characterization of CsO/IGA

Nanomaterials have emerged as promising therapeutic carriers due to their ability to enhance permeability and overcome biological barriers, thereby maximizing anticancer potential [28]. Nanocarrier CsO was synthesized following our previously reported method [25]. Initial characterization of CsO NPs revealed a hydrated particle size of approximately 160 nm, a zeta potential of −16.1 mV, and uniform size distribution (Table S1 and Fig. 1D). To identify the optimal formulation, we characterized CsO loaded with varying weight ratios of single or dual drugs (Tables S1–S3). The particle size of CsO/A showed varying degrees of increase after drug loading (Table S1). Given the need for subsequent dual-drug loading, we prioritized smaller particle size formulations. The EE of AZD9291 decreased progressively with higher loading, suggesting limited CsO capacity for AZD9291. Although CsO/A (200:1) exhibited the highest EE, its larger particle size was suboptimal for delivery. Additionally, because AZD9291 requires a threshold concentration for pharmacological efficacy, the weight ratio of CsO:AZD9291 was ultimately set at 200:2 for subsequent studies.

Characterization of CsO/IG prepared at different CsO:ICG weight ratios (Table S2) revealed minimal EE variations, which could be attributable to Gd^3+^-enhanced electrostatic interactions between CsO and ICG-Gd. When the weight ratio was 200:40, the nanocomplexes demonstrated the smallest particle size, a relatively high absolute zeta potential value, and favorable EE. Consequently, the 200:40 weight ratio was selected as the optimal formulation for CsO/IG.

Considering the limited capacity of CsO for loading dual drugs, CsO/IGA ratios were also screened (Table S3). The formulation with 200:40:2 ratio exhibited optimal characteristics including approximately 100 nm diameter, minimal PDI, and the highest absolute zeta potential value, indicating superior stability. While both 200:40:2 and 200:50:2 ratios achieved high EE for both ICG-Gd and AZD9291, the latter showed excessive PDI and heterogeneous nanoparticle size distribution. Consequently, the 200:40:2 ratio was selected for CsO/IGA preparation in subsequent studies.

Optimized formulations were thoroughly characterized (Fig. 1D–G). CsO/A, CsO/IG, and CsO/IGA all exhibited uniform size distribution. The zeta potentials were all negative, with CsO/IG and CsO/IGA showing increased absolute values compared to CsO NPs, indicating that ICG-Gd loading could enhance stability against aggregation. TEM results revealed that both CsO NPs (Fig. S2) and CsO/IGA (Fig. 1F) displayed regular spherical morphologies. CsO/IG and CsO/IGA showed characteristic absorption peaks of ICG at 780 nm, confirming successful loading of ICG-Gd. Similarly, CsO/A and CsO/IGA exhibited characteristic peaks of AZD9291 at 317 nm, demonstrating successful incorporation of AZD9291 (Fig. 1G). In contrast, the formulation without OA modification (Cs/IGA) was unstable and formed immediate precipitates upon preparation (Fig. S3), which precluded its use in further biological evaluations.

### 3.3 Stability and release properties of CsO/IGA

The stability of CsO/IGA was systematically evaluated by monitoring particle size distribution (Fig. S4A), hydrodynamic diameter (Fig. 1H), and PDI (Fig. S4B) over 7 days in various media. CsO/IGA exhibited uniform particle size distribution in ddH_2_O and PBS, maintaining a size of around 100 nm for 7 days. In ddH_2_O, the PDI remained stable, while a slight increase was observed in PBS at day 5, but the dispersibility remained acceptable for injectable applications. When dispersed in RPMI-1640 and RPMI-1640 + 10% FBS, CsO/IGA retained its nanoscale characteristics, but an additional small peak appeared in the size distribution profile, likely due to the interactions with high concentrations of vitamins and proteins in RPMI-1640 and FBS. These results confirm adequate stability of CsO/IGA for cellular applications, though fresh preparation is recommended prior to use. Zeta potential measurements revealed sustained colloidal stability over 5 days, with high absolute values indicating effective electrostatic stabilization (Fig. 1I). However, the decreased zeta potential at day 7 suggested diminished interparticle repulsion and potential aggregation risk. Therefore, to ensure optimal dispersibility, CsO/IGA should be stored and used within one week.

The triterpenoid- and Cs-based drug delivery systems demonstrated well-defined pH-responsive release characteristics [29], showing significantly enhanced drug release in tumor-mimicking acidic conditions (pH 5.5) compared to physiological conditions (pH 7.4). Using 20% ethanol-PBS (pH 7.4 or 5.5) as the release medium, the release behaviors of ICG and AZD9291 from CsO/IGA were investigated with or without laser irradiation (Fig. 1J and K). CsO/IGA remained stable under physiological conditions, indicating favorable biosafety profiles. Laser irradiation significantly enhanced ICG release, with the most dramatic effect observed under the combined conditions of acidic pH and laser stimulation, suggesting the potential for synergistic phototherapeutic applications. While laser exposure had minimal impact on AZD9291 release, both therapeutic agents showed markedly increased release rates in acidic environments, confirming the pH-responsive drug delivery capability of CsO/IGA for targeted tumor therapy.

### 3.4 Functional evaluation of CsO/IGA for theranostic applications

The FLI/MRI dual-imaging ability of ICG-Gd-based nanocomplexes were characterized *in vitro* (Fig. 1L and M). ICG-Gd exhibited lower fluorescence intensity than free ICG but retained FLI capability. Both CsO/IG and CsO/IGA demonstrated further reduced fluorescence intensity compared to ICG-Gd, likely due to aggregation-caused quenching resulting from the encapsulation of ICG-Gd within the nanocomplexes. In MRI, all three ICG-Gd-based formulations exhibited higher MRI contrast than the clinical contrast agent Gd-DOTA, suggesting their potential as superior MRI contrast agents. The lower FLI and MRI signals of CsO/IG and CsO/IGA compared to free ICG-Gd can be attributed to nanocomplex stability in aqueous solution, preventing ICG-Gd release. Compared with CsO/IG, CsO/IGA displayed higher MRI signal intensity. This is attributed to the incorporation of AZD9291, which induces physicochemical changes within the nanoplatform. These changes likely optimize the dispersion state of ICG-Gd and increase the accessibility of surrounding water molecules to the Gd^3+^ centers, thereby leading to higher *T*_*1*_ relaxivity and stronger contrast. These results confirm that ICG-Gd-based contrast agents possess excellent FLI/MRI dual-modality imaging capabilities.

**Fig. 2:**
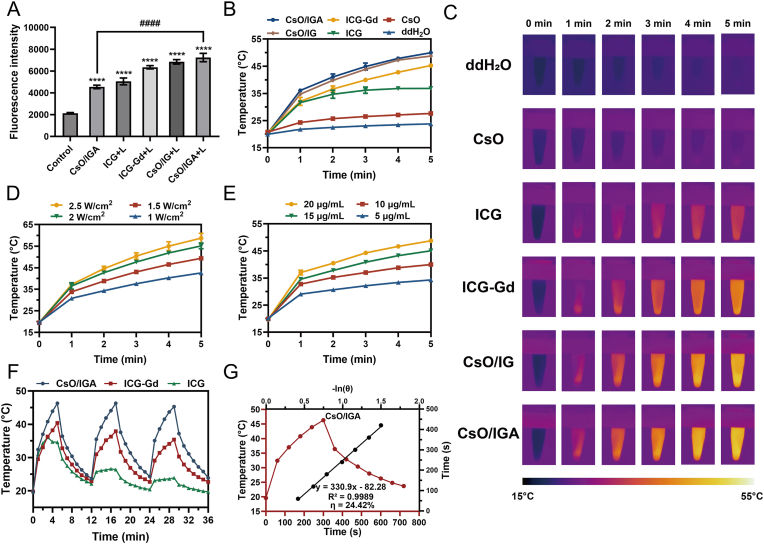
^1^O_2_ generation capability and photothermal properties of CsO/IGA. (A) ^1^O_2_ generation capacity of different samples quantified by the fluorescence intensity of SOSG probe. L: laser irradiation group (808 nm, 2 W/cm^2^, 3 min). ∗∗∗∗p < 0.0001 compared with Control group. ####p < 0.0001 between indicated groups. (B-C) Temperature changes and corresponding photothermal images of ddH_2_O, CsO, ICG, ICG-Gd, CsO/IG, and CsO/IGA (containing 15 μg/mL ICG) under 808 nm laser irradiation (1.5 W/cm^2^). (D) Temperature changes of CsO/IGA (containing 15 μg/mL ICG) under 808 nm laser irradiation at different power densities for 0-5 min. (E) Temperature elevation of CsO/IGA at various ICG concentrations under 808 nm laser irradiation (1.5 W/cm^2^) for 0-5 min. (F) Temperature changes of ICG, ICG-Gd, and CsO/IGA during three alternating ON/OFF laser irradiation cycles (808 nm, 1.5 W/cm^2^). (G) Temperature-time curves (red line) and cooling period-derived time constants (black line) for calculating the PCE of CsO/IGA based on the first cycle in Fig. 2F. (For interpretation of the references to colour in this figure legend, the reader is referred to the Web version of this article.)

The generation of ^1^O_2_ by various samples under 808 nm laser irradiation was detected using the SOSG fluorescent probe (Fig. 2A). CsO/IGA produced a certain amount of ^1^O_2_ even without laser irradiation, while the ^1^O_2_ production significantly increased with laser irradiation, demonstrating its laser-dependent photodynamic effect. Under laser irradiation, ICG could convert light energy into ^1^O_2_ through the type II PDT mechanism. The formation of the ICG-Gd complex effectively enhanced the stability of ICG, resulting in higher ^1^O_2_ production. Encapsulation within the CsO nanocarrier further improved stability, allowing CsO/IG and CsO/IGA to generate more ^1^O_2_ than free ICG-Gd. Thus, the combination of laser irradiation and nanostructured carriers synergistically enhanced the photodynamic activity of CsO/IGA.

The photothermal performance of the samples was evaluated by monitoring temperature changes under laser irradiation using an infrared thermal camera. Fig. 2B and C presents the temperature-time curves and thermal images of ddH_2_O, CsO, ICG, ICG-Gd, CsO/IG, and CsO/IGA. Neither ddH_2_O nor CsO exhibited photothermal properties, with only a minor temperature increase due to the inherent thermal effect of the 808 nm laser itself. Free ICG showed rapid temperature rise within 1 min, followed by a plateau due to photodegradation. In contrast, the complexation of Gd^3+^ in ICG-Gd helped maintaining the structural stability of ICG under laser irradiation, enabling sustained temperature rise. When encapsulated in the CsO nanocarrier, both CsO/IG and CsO/IGA reached higher maximum temperatures after 5 min of laser irradiation. CsO/IGA demonstrated significant time-dependent heating, with temperature increases positively correlated with laser power intensity (Fig. 2D) and ICG concentration (Fig. 2E).

Photothermal stability was evaluated through three laser ON/OFF cycles (Fig. 2F). ICG showed rapid photodegradation, with temperature decline after 3 min in the first cycle. In contrast, both ICG-Gd and CsO/IGA maintained heating capability throughout three cycles, demonstrating excellent photothermal stability. Notably, while ICG-Gd exhibited peak temperature reduction in each cycle, CsO/IGA maintained nearly consistent peak temperatures, confirming the protective role of the CsO nanocarrier. The PCE (η) calculated from the first cycle data was 10.93% for ICG, 16.25% for ICG-Gd, and 24.42% for CsO/IGA (Fig. S5 and Fig. 2G). These results clearly demonstrate that both Gd^3+^ complexation and CsO encapsulation significantly enhance the PCE of ICG.

CsO/IGA demonstrated an excellent FLI/MRI dual-modality imaging, as well as remarkable temperature elevation and sustained ^1^O_2_ generation under laser irradiation, indicating that the encapsulation of ICG-Gd by CsO not only enhanced the structural stability of ICG-Gd but also significantly improved its combined photodynamic and photothermal therapeutic effects, demonstrating considerable potential for theranostic applications.

### 3.5 Development of AZD9291-resistant NSCLC cell lines with graduated resistance

Gradually increasing drug concentrations resulted in progressively higher viability of AZD9291-resistant cell lines (P1-P6) when treated with AZD9291 (Fig. S6), indicating reduced cytotoxic efficacy of AZD9291. These results confirmed the successful establishment of PC9-derived resistant cell models with varying degrees of AZD9291 resistance, demonstrating significantly reduced drug sensitivity.

To identify the most suitable resistant cell line for further studies, CsO/IGA uptake in P1-P6 cells was evaluated by flow cytometry (Fig. S7). Despite acquired resistance, all cell lines retained significant nanoparticle internalization via endocytosis. However, P1-P4 cells showed reduced CsO/IGA uptake compared to parental PC9 cells, suggesting partial resistance to nanoparticle entry. In contrast, P6 cells exhibited enhanced uptake, likely due to membrane permeability alterations induced by prolonged high-dose AZD9291 exposure [30]. Notably, while certain resistant cells maintained efficient nanoparticle internalization, prior studies indicated that the resistant cells may sequester nanoparticles into lysosomes or other inactive vesicular structures to attenuate therapeutic effects [31]. Given that P6 cells displayed compromised physiological stability from chronic high-dose AZD9291 induction, we ultimately selected P5 cells which maintained close nanoparticle uptake to PC9 cells for subsequent experiments to ensure experimental robustness. Notably, while the P5 cell line exhibits a stable resistance phenotype, its genetic basis (such as EGFR T790M/C797S mutations or MET amplification) has not yet been characterized. Whether the resistance is on-target (EGFR dependent) or off-target (bypass activation) remains to be determined.

### 3.6 Cellular uptake of CsO/IGA by different subtypes of NSCLC cells

**Fig. 3:**
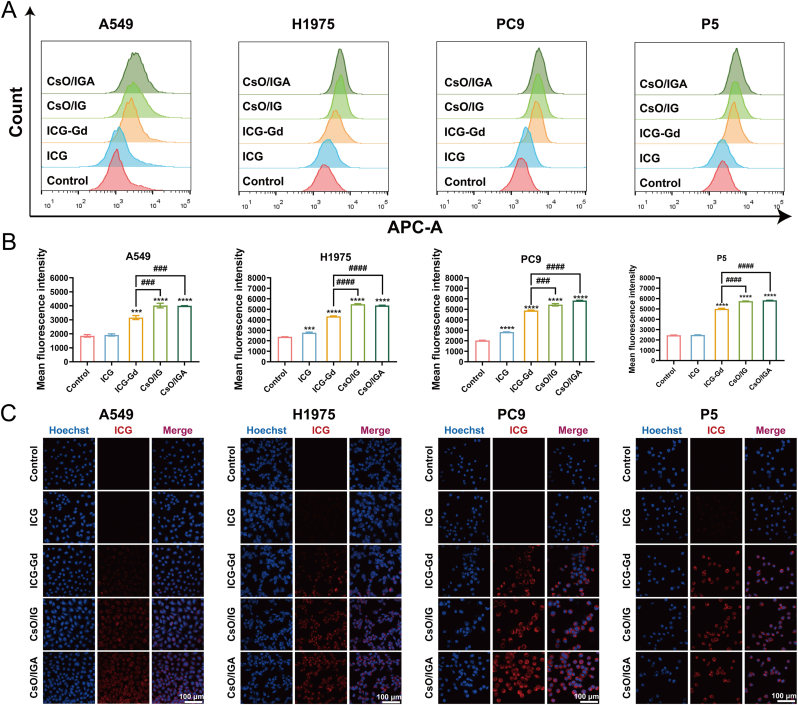
*In vitro* cellular uptake efficiency. (A) Flow cytometry histograms and (B) mean fluorescence intensity of A549, H1975, PC9, and P5 cells treated with ICG, ICG-Gd, CsO/IG, and CsO/IGA for 4 h ∗∗∗p < 0.001, ∗∗∗∗p < 0.0001 compared with Control group. ###p < 0.001, ####p < 0.0001 between indicated groups. (C) CLSM images showing the intracellular distribution of ICG, ICG-Gd, CsO/IG, and CsO/IGA in A549, H1975, PC9, and P5 cells after 4 h treatment. Cell nuclei are stained blue (Hoechst 33342), and red fluorescence represents ICG signal from different drug formulations. Scale bars = 100 μm. (For interpretation of the references to colour in this figure legend, the reader is referred to the Web version of this article.)

The cellular uptake of CsO/IGA was evaluated in three NSCLC cell subtypes—A549 (EGFR wild-type, KRAS mutation) [32,33], H1975 (EGFR T790M/L858R mutation) [34], and PC9 (EGFR exon 19 deletion) [34]. Flow cytometry analysis (Fig. 3A and B) showed significantly increased cellular fluorescence intensity following 4 h incubation with ICG-Gd, CsO/IG, and CsO/IGA compared to control groups, confirming efficient cellular internalization of all three formulations. The Gd^3+^ complexation caused a certain degree of fluorescence quenching in ICG but enhanced the fluorescence stability of ICG. Moreover, Gd^3+^ was found to increase cell membrane permeability upon cellular entry [35], thereby facilitating improved ICG-Gd uptake by NSCLC cells. When ICG-Gd was loaded onto CsO nanocarriers, the resulting CsO/IG and CsO/IGA nanocomplexes showed further enhanced cellular uptake compared to free ICG-Gd, which is attributable to both improved fluorescence stability and more efficient nanoparticle endocytosis [36].

CLSM observations corroborated the flow cytometry results (Fig. 3C). Blue fluorescence indicates the cell nucleus, while red fluorescence represents ICG signals from internalized drug formulations. The CLSM images revealed predominant cytoplasmic distribution of all formulations following cellular internalization. CsO/IG and CsO/IGA exhibited substantial cellular uptake even in AZD9291-insensitive A549 cells, consistent with previous reports that nanocarriers can enhance drug accumulation in tumor cells through improved stability and cellular internalization mechanisms [37].

Similarly, flow cytometry and CLSM were employed to evaluate the cellular uptake of P5 cells (Fig. 3). Consistent with the results in PC9 cells, the Gd^3+^ complexation significantly enhanced ICG-Gd uptake compared to free ICG, while CsO encapsulation further improved stability and cellular internalization. CLSM images revealed that ICG-Gd, CsO/IG and CsO/IGA were primarily distributed in the cytoplasm of P5 cells with minimal nuclear accumulation.

### 3.7 *In vitro* combination therapeutic efficacy and action mechanisms of CsO/IGA in reversing EGFR-TKI resistance

Given the significant proportion of CsO carrier in the nanodrug formulation, its cytotoxicity was first evaluated (Fig. S8). Due to the anti-cancer effects of OA [38], the proliferation inhibition effect gradually increased with rising CsO concentrations in A549, H1975, PC9, and P5 cells. However, since high CsO concentrations may also affect normal cells [25], careful optimization of CsO concentration was required for subsequent experiments to balance sufficient drug loading capacity and synergistic anti-tumor efficacy while minimizing potential toxicity. Comparative cytotoxicity assessment between free AZD9291 and CsO/A (Fig. S9) revealed stronger cytotoxicity of CsO/A against H1975 and PC9 cells, suggesting enhanced therapeutic efficacy through improved drug delivery efficiency in NSCLC cells. AZD9291 showed weak cytotoxicity against target-deficient A549 cells and resistant P5 cells. In contrast, CsO/A demonstrated concentration-dependent proliferation inhibition in A549 cells, indicating the ability of CsO delivery carrier to enhance drug potency against targeted therapy-insensitive tumors. The increased cytotoxicity of CsO/A against P5 cells further suggested that CsO delivery could improve drug sensitivity in resistant cells, demonstrating potential resistance reversal capability.

**Fig. 4:**
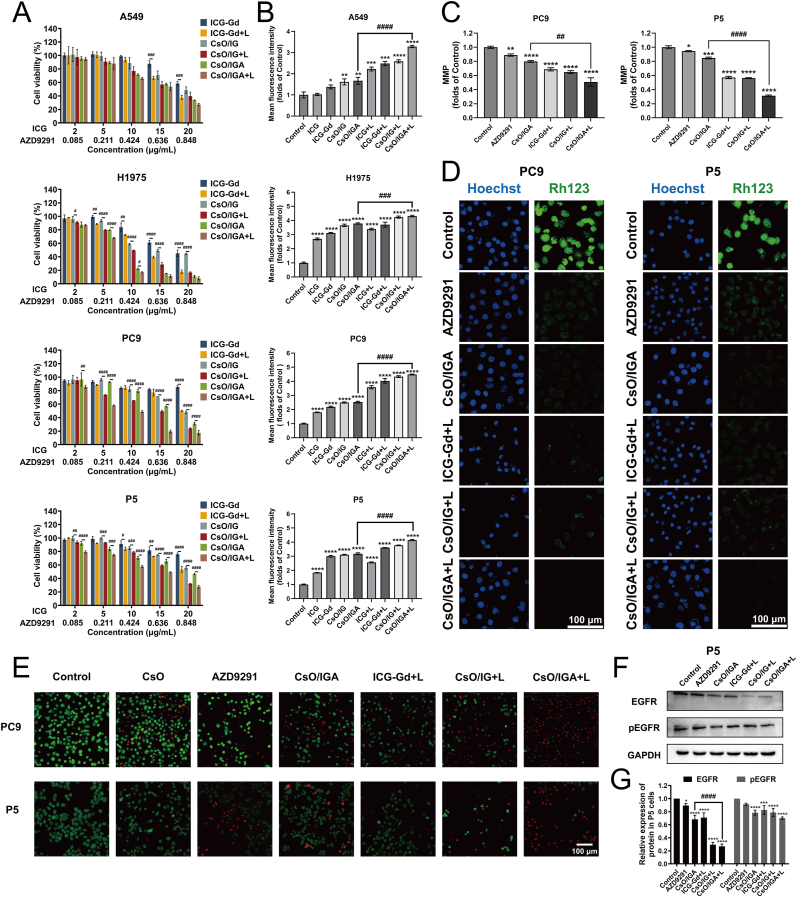
*In vitro* synergistic therapeutic effects, anti-cancer and drug-resistance reversal mechanisms of CsO/IGA. CsO/IGA combined with laser irradiation demonstrated superior anti-cancer efficacy across all cell lines, including enhanced cytotoxicity, increased ROS production, MMP loss, effective cell death, and reversal of drug resistance via EGFR/p-EGFR downregulation. (A) Cell viability of A549, H1975, PC9, and P5 cells treated with ICG-Gd, CsO/IG, and CsO/IGA at equivalent ICG concentrations for 24 h with or without laser irradiation. #p < 0.05, ##p < 0.01, ###p < 0.001, ####p < 0.0001 compared with non-irradiation group. (B) Flow cytometry analysis of ROS production in A549, H1975, PC9, and P5 cells after different treatments. (C) Flow cytometry analysis and (D) CLSM images of MMP changes in PC9 and P5 cells after different treatments. (E) Live/dead cell staining assay for PC9 and P5 cells after different treatments. (F) Western blot analysis of EGFR and p-EGFR expressions in P5 cells after different treatments. (G) Semi-quantitative analysis of relative protein expression levels in P5 cells. L: laser irradiation (808 nm, 1 W/cm^2^, 1 min). ∗p < 0.05, ∗∗p < 0.01, ∗∗∗p < 0.001, ∗∗∗∗p < 0.0001 compared with Control group. ##p < 0.01, ###p < 0.001, ####p < 0.0001 between indicated groups.

As a NIR-responsive phototherapeutic agent, ICG can generate ROS and heat upon cellular uptake, enabling synergistic PDT/PTT effects to inhibit tumor proliferation [20]. Studies have found that complexing ICG with metal ions such as Fe^3+^ and Au^2+^ enhances its NIR absorption capacity and photostability, leading to improved phototherapeutic efficacy [22,39]. It has demonstrated that ICG-Gd-based formulations exhibit potential for PDT/PTT applications under laser irradiation (Fig. 2). The optimal laser irradiation condition for cellular experiments through MTT assays were determined to be 1 W/cm^2^ for 1 min (Fig. S10). To evaluate the synergistic antitumor efficacy, the cytotoxicity of ICG-Gd, CsO/IG, and CsO/IGA was assessed under laser irradiation (Fig. 4A). High-concentration ICG-Gd exhibited slight toxicity, indicating that Gd^3+^ has minimal impact on the cytotoxicity of ICG. Under laser irradiation, ICG-Gd demonstrated concentration-dependent PDT/PTT efficacy. Both CsO/IG and CsO/IGA exhibited stronger proliferation inhibition in A549 cells compared to ICG-Gd, confirming the effectiveness of CsO combination. Due to the high sensitivity of H1975 cells to AZD9291, CsO/IGA significantly inhibited the cell viability regardless of laser irradiation. In PC9 cells, increasing concentrations of CsO/IG and CsO/IGA progressively reduced viability, with CsO/IGA showing superior cytotoxicity at concentrations ≥10 μg/mL. The CsO encapsulation strategy shielded AZD9291 from recognition by P5 cells while leveraging nanoparticle endocytosis to enhance drug uptake in resistant cells, resulting in significantly stronger cytotoxicity of CsO/IGA against P5 cells compared to CsO/IG and ICG-Gd. For both PC9 and P5 cells, the CsO/IGA+L group showed lower cell viability than CsO/IGA group, further confirming the synergistic chemo-phototherapeutic effect of CsO/IGA.

The photodynamic action mechanisms of CsO/IGA were systematically investigated through comparative analysis of ROS generation under various treatment conditions (different drug formulations with or without laser irradiation) in A549, H1975, PC9, and P5 cells (Fig. 4B). ICG-Gd, CsO/IG, and CsO/IGA treatments all induced significantly elevated ROS production compared to free ICG, with the nanoparticle formulations showing particularly pronounced effects. Following 808 nm laser irradiation, all treatment groups exhibited substantially increased ROS levels relative to non-irradiated controls, confirming the laser-dependent ROS generation. The combination of nanocomplex-mediated enhanced cellular accumulation and precise laser stimulation resulted in markedly elevated intracellular ROS concentrations, ultimately leading to remarkable enhanced PDT efficacy. Complementary CLSM observations in these four cell lines revealed a positive correlation between green fluorescence intensity and ROS levels (Figs. S11–S14). The results confirmed that effective ROS production requires both efficient cellular uptake of nanodrugs and appropriate laser stimulation, consistent with quantitative flow cytometry data. PC9 and P5 cells exhibited similar patterns of ROS generation with ICG-Gd, CsO/IG, and CsO/IGA treatments under both laser and non-laser conditions. The parallel response patterns observed in PC9 cells and their drug-resistant P5 counterparts across all treatment conditions validated these cell models for investigating the antitumor mechanisms of the nanodrug system. Therefore, these two cell lines were selected for subsequent experimental studies.

Further investigation of MMP alterations in PC9 and P5 cells showed treatment-dependent patterns of mitochondrial damage (Fig. 4C). AZD9291 treatment induced MMP reduction in sensitive PC9 cells, consequently leading to cytotoxic effects. CsO/IGA further aggravated mitochondrial impairment in PC9 cells, with laser irradiation further exacerbating this effect. Due to acquired drug resistance, AZD9291 exhibited weaker MMP inhibition in resistant P5 cells compared to PC9 cells. Notably, CsO/IGA with laser irradiation caused the most substantial mitochondrial damage in P5 cells, demonstrating that CsO/IGA could overcome drug resistance through synergistic chemo-photodynamic action. CLSM results demonstrated a significant reduction in green fluorescence intensity in PC9 and P5 cells under drug treatments, indicating effective decrease in MMP (Fig. 4D). CsO/IGA+L group showed the most pronounced fluorescence attenuation, which is consistent with the flow cytometry results. These findings further indicate that the combined chemo-photodynamic therapy can significantly enhance mitochondrial damage in both EGFR-TKIs-sensitive and resistant cell lines, providing mechanistic insights into its superior anticancer efficacy.

The Calcein-AM/PI double staining method was further used to differentially labels live cells (displaying bright green fluorescence) and dead cells (exhibiting red fluorescence) to assess the combination efficacy of CsO/IGA on PC9 and P5 cells. Quantitative analysis revealed a marked increase in red fluorescence intensity in cells treated with CsO/IGA compared to control groups (Fig. 4E). After laser irradiation, the population of red-fluorescent cells predominated over green-fluorescent cells, indicating that the combination of CsO/IGA with photothermal stimulation could induce highly efficient cell necrosis. These results provide visual evidence of the superior synergistic therapeutic effect achieved through the combined chemo-photothermal treatment approach.

The most common resistance mechanisms to AZD9291 involve EGFR-dependent pathways, including secondary EGFR mutations and gene amplification events. The hyperactivation of EGFR leads to the production of pEGFR protein, which subsequently activates downstream proliferative signaling cascades in resistant cells [40]. To evaluate the resistance-reversal potential of our therapeutic approach, we examined EGFR and pEGFR protein expression profiles in P5 cells (Fig. 4F and G). All tested formulations demonstrated stronger suppression of total EGFR expression than pEGFR, suggesting dual inhibitory effects on both the pEGFR signaling pathway and EGFR gene amplification. The CsO/IGA nanocomplex demonstrated particular efficacy by functionally masking AZD9291 from recognition by resistant cells while simultaneously enhancing intracellular drug delivery, resulting in significant downregulation of both EGFR and pEGFR expression. The most substantial suppression of EGFR/pEGFR proteins occurred in the CsO/IG+L and CsO/IGA+L treatment groups. To further elucidate the downstream signaling consequences of EGFR inhibition, we examined total AKT expression levels across different treatment groups (Fig. S15). Compared with the control group and the AZD9291 monotherapy group, total AKT expression levels were significantly reduced in the CsO/IG+L and CsO/IGA+L groups. This result confirms that the combined CsO/IGA treatment suppresses downstream AKT signaling and further clarifies the molecular mechanism responsible for EGFR-TKI resistance reversal. Collectively, these findings clearly establish that the integrated nanoparticle-mediated chemo-photothermal therapy can simultaneously target multiple resistance mechanisms, providing a robust strategy to overcome EGFR-TKI resistance.

### 3.8 Regulation of CAFs

As a key component of the TME, CAFs construct a microenvironment conducive to tumor progression by continuously secreting and releasing growth factors and cytokines, thereby promoting tumor cell survival, therapy evasion, and drug resistance [41]. The aberrant activation of TGF-β1 induces the differentiation of normal fibroblasts into α-SMA^+^ CAFs [41]. Immunofluorescence analysis confirmed that 3T3^+^ cells exhibit a distinct fibrotic morphology and express elevated levels of α-SMA, which is the most widely recognized biomarker for identification and characterization of CAFs. Thus, these cells serve as a suitable model for studying α-SMA-high-expressing CAFs (Fig. S16). Previous studies have demonstrated that OA exhibits potent TME-regulatory functions, typically achieved through nanomedicine-based delivery systems [42]. However, it remains uncertain whether chemically conjugating OA into drug delivery systems preserves its TME-regulatory effects. To verify whether CsO and CsO-based nano-formulations retain antifibrotic effects to regulate the TME, 3T3^+^ cells with CAF-like characteristics were established.

**Fig. 5:**
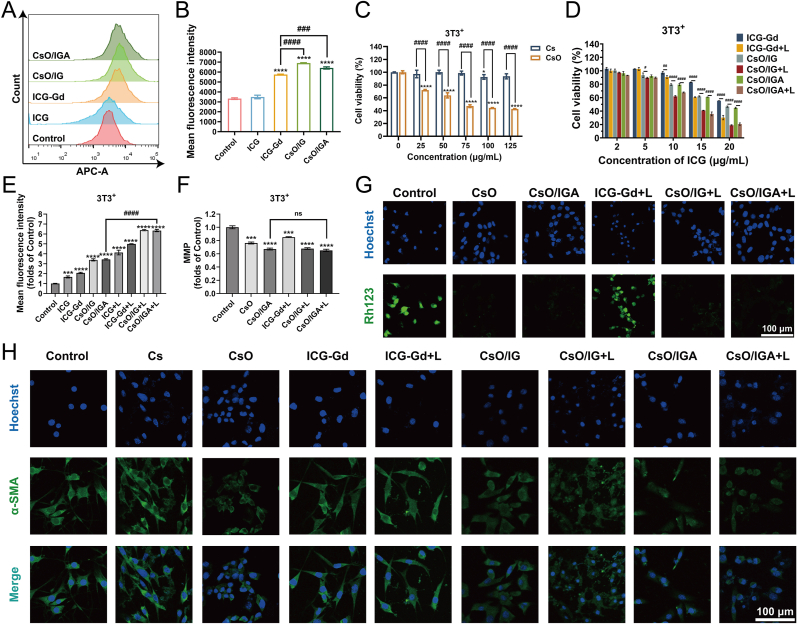
Regulatory effects on CAFs. CsO/IGA combined with laser irradiation exhibited the most potent effects on CAFs, including efficient cellular uptake, reduced cell viability, increased ROS production, MMP loss, and downregulation of α-SMA expression. (A) Flow cytometry histograms and (B) mean fluorescence intensity of 3T3^+^ cells treated with ICG, ICG-Gd, CsO/IG, and CsO/IGA for 4 h. (C) Viability of 3T3^+^ cells treated with Cs or CsO for 48 h. (D) Viability of 3T3^+^ cells treated with ICG-Gd, CsO/IG, or CsO/IGA for 24 h with or without laser irradiation. (E) Flow cytometry analysis of ROS production in 3T3^+^ cells after different treatments. (F) Flow cytometry analysis of MMP changes in 3T3^+^ cells stained with Rh 123 after different treatments. (G) CLSM images showing MMP changes in 3T3^+^ cells after different treatments. (H) Immunofluorescence analysis of α-SMA expression in 3T3^+^ cells. L: laser irradiation (808 nm, 1 W/cm^2^, 1 min). ∗∗∗p < 0.001, ∗∗∗∗p < 0.0001 compared with Control group. ns: not significant, #p < 0.05, ##p < 0.01, ###p < 0.001, ####p < 0.0001 between indicated groups.

Cell uptake experiments (Fig. 5A and B) revealed substantial ICG-Gd accumulation in 3T3^+^ cells, likely due to Gd^3+^-mediated modulation of cell membrane permeability. When loaded into CsO, the nanocomplexes exhibited significantly enhanced cellular uptake compared to free ICG-Gd. These findings preliminarily confirm that the CsO nanocarrier can effectively promote drug accumulation in CAFs, providing an experimental basis for further studies on CsO-mediated CAF regulation.

At low concentrations, CsO alone effectively inhibited the growth of 3T3^+^ cells (Fig. 5C), demonstrating that OA conjugation preserved its CAF-regulating function. Both CsO/IGA and CsO/IG exhibited comparable effects on 3T3^+^ cells (Fig. 5D). The CsO control group at the corresponding concentration range (Fig. S17) showed similar effects, further suggesting that CsO is the primary active component responsible for regulating CAF activity. Furthermore, the cytotoxicity increased significantly after laser irradiation, indicating that the combined PDT/PTT effect contributed substantially to the suppression of CAF activity.

Compared to ICG-Gd, 3T3^+^ cells treated with CsO/IG and CsO/IGA generated significantly higher levels of ROS, confirming that CsO enhances intracellular delivery of ICG-Gd into 3T3^+^ cells and facilitates efficient ROS production upon laser irradiation, thereby suppressing fibroblast function (Fig. 5E). Although ICG-Gd alone could induce ROS generation and hyperthermia under laser irradiation, its mitochondrial damage effect in 3T3^+^ cells was notably weaker than that of the CsO-based formulations (Fig. 5F and G). Treatment with CsO-based nanocomplexes also led to a pronounced decrease in MMP, further supporting the dominant role of CsO in suppressing CAF activity. Interestingly, laser-induced mitochondrial damage was negligible in 3T3^+^ cells, whereas a pronounced effect was observed in PC9 and P5 cells (Fig. 4C). This cell-type-dependent difference can be attributed to differential regulatory mechanisms between CAFs and cancer cells. Specifically, CAFs are highly sensitive to the OA component of CsO, such that the additional phototherapeutic effect from laser irradiation does not further enhance mitochondrial damage. In contrast, PC9 and P5 cancer cells are less sensitive to OA (Fig. S7), making the laser-induced phototherapeutic effect a major contributor to their mitochondrial injury.

The antifibrotic effects were evaluated by examining α-SMA expression in 3T3^+^ cells (Fig. 5H). CsO treatment significantly reduced green fluorescence intensity, indicating strong suppression of α-SMA. In contrast, formulations lacking OA modification (Cs, ICG-Gd, and ICG-Gd+L) failed to reduce α-SMA fluorescence, highlighting the critical role of OA-modified Cs in remodeling the TME. While CsO/IG and CsO/IGA also reduced α-SMA expression, the minimal difference between laser- and non-laser-treated groups suggested that the antifibrotic effect of CsO/IGA was primarily mediated by CsO rather than phototherapy. These results demonstrated that CsO/IGA effectively inhibited CAF proliferation and α-SMA expression, thereby suppressing fibroblast activation and potentially enabling deeper tumor penetration.

### 3.9 Deep penetration and *in vivo* FLI/MRI

**Fig. 6:**
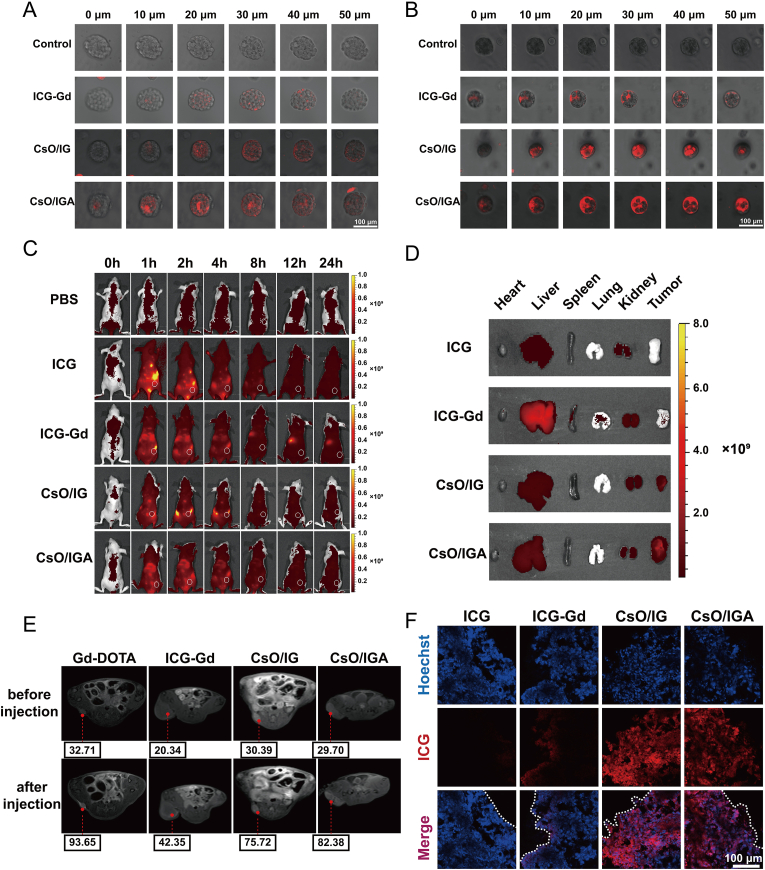
Tumor penetration and *in vivo* FLI/MRI. Penetration of CsO/IGA into (A) PC9 and (B) P5 3D tumor spheroids after 4 h incubation. (C) Time-course *in vivo* FLI of P5 tumor-bearing mice after intravenous injection. (D) *Ex vivo* FLI of excised tumors and major organs after 2 h of injection. (E) *In vivo* transverse MRI of P5 tumor-bearing mice before and after injection. (F) CLSM images of frozen tumor slices from P5 tumor-bearing mice after 2 h of injection (white dotted lines indicate tumor margins). Scale bar = 100 μm. Red fluorescence represents ICG. (For interpretation of the references to colour in this figure legend, the reader is referred to the Web version of this article.)

Monolayer cell cultures often lose tissue-specific characteristics, whereas 3D tumor models can better emulate the properties of native tissues, making them more physiologically relevant for evaluating nanotherapeutic responses [43]. Given that CsO/IGA demonstrated antifibrotic effects which may enhance drug penetration by disrupting fibrotic barriers, we assessed its penetration capacity through ICG red fluorescence signals using PC9 and P5 cell-derived 3D tumor spheroids (Fig. 6A and B). Weak fluorescence signals were primarily distributed at the periphery of 3D tumor spheroids treated with ICG-Gd, indicating limited penetration depth despite enhanced cellular uptake. In contrast, both CsO/IG and CsO/IGA effectively delivered drugs into the core regions of 3D tumor spheroids with homogeneous ICG fluorescence distribution throughout the entire spheroid. These results demonstrated the superior penetration capacity of CsO-based nanocomplexes, showing promise for achieving more comprehensive and rapid therapeutic effects.

Prior to *in vivo* studies, hemolysis assay was conducted to preliminarily evaluate the biosafety of CsO/IGA and provide a reference dosage for *in vivo* administration (Fig. S18). CsO/IGA exhibited good hemocompatibility at concentrations below 15 μg/mL. Therefore, a dosage of 15 μg/mL was set as the upper threshold for subsequent *in vivo* dosing.

Following administration in P5 tumor-bearing mice, we dynamically monitored fluorescence signals using a small animal imaging system (Fig. 6C) and quantified the fluorescence intensity over time (Fig. S19). All three ICG-Gd-based formulations demonstrated significant fluorescence enhancement, confirming their capability for *in vivo* FLI. At 2 h post-administration, while ICG-Gd maintained stable fluorescence signals, both CsO/IG and CsO/IGA showed peak fluorescence intensity, with all ICG-Gd formulations exhibiting prolonged circulation compared to rapidly cleared free ICG. In cancer treatment, the accumulation of drugs at the tumor site is a prerequisite for exerting antitumor efficacy [44]. To better characterize intratumoral distribution patterns, we conducted *ex vivo* tissue analysis at 2 h post-administration (Fig. 6D). CsO/IG and CsO/IGA revealed significantly greater tumor accumulation compared to ICG-Gd, which could be due to the enhanced permeation and retention (EPR) effects [45]. The findings established the 2-h timepoint as optimal for subsequent laser irradiation therapy. Importantly, all ICG-Gd-based formulations showed efficient clearance through hepatic and renal pathways, thereby minimizing potential systemic toxicity concerns.

MRI evaluation of subcutaneous tumors using Gd-DOTA as a positive control revealed distinct contrast enhancement patterns (Fig. 6E). While Gd-DOTA exhibited 2.86-fold enhancement, the enhancement was primarily confined to the tumor periphery, lacking the ability to penetrate deep into the tumor tissue. ICG-Gd, CsO/IG, and CsO/IGA showed 2.08-fold, 2.49-fold, and 2.77-fold tumor tissue contrast enhancements after injection, respectively. Notably, CsO/IGA demonstrated homogeneous contrast throughout tumor tissues, suggesting both enhanced diagnostic accuracy and superior deep-tumor penetration capability of CsO-based nanoformulations. To provide objective quantitative support for the qualitative MRI observations, we performed signal-to-noise ratio (SNR) analysis of the tumor region before and after administration of each probe. As shown in Fig. S20, the baseline SNR values were comparable across all groups before injection, while post-injection SNR was notably elevated in all cases. Specifically, the SNR increased from 1.51 ± 0.05 to 2.20 ± 0.06 in the Gd-DOTA, from 1.32 ± 0.06 to 1.46 ± 0.02 in the ICG-Gd, from 1.34 ± 0.03 to 1.59 ± 0.03 in the CsO/IG, and from 1.28 ± 0.07 to 1.75 ± 0.06 in the CsO/IGA. Collectively, these data confirm that all nanoformulations can effectively enhance MRI signals at the tumor site, validating their imaging enhancement capabilities.

To achieve therapeutic efficacy, nanomedicines must overcome multiple biological barriers including circulation, tumor accumulation, deep penetration, and intracellular release [46]. Among these, the obstacle of deep tumor penetration arises from poor drug permeation, which prevents drugs from reaching the depths of tumor tissue far from blood vessels, thereby reducing therapeutic efficacy [47]. To further evaluate the deep tumor penetration capability of the nanocomplexes, tumor tissues were cryosectioned and analyzed using CLSM (Fig. 6F). Consistent with the *in vitro* 3D tumor spheroid results, ICG-Gd was primarily distributed at the periphery of the solid tumor, while CsO/IG and CsO/IGA exhibited strong red fluorescence which was clearly observed in deep inside of tumor tissue. This indicated that CsO-based nano-formulations possessed deep tumor penetration capability.

### 3.10 *In vivo* anti-tumor efficacy

The *in vivo* dose of CsO/IGA was determined based on preliminary efficacy studies showing optimal tumor inhibition without systemic toxicity. Although this dose exceeded the maximum concentration tested in the hemolysis assay, the latter represents a static worst-case *in vitro* scenario with direct and prolonged contact with red blood cells. In contrast, *in vivo* administration involves rapid blood dilution, tissue distribution, and clearance, resulting in substantially lower actual blood concentrations and minimal hemolysis risk.

**Fig. 7:**
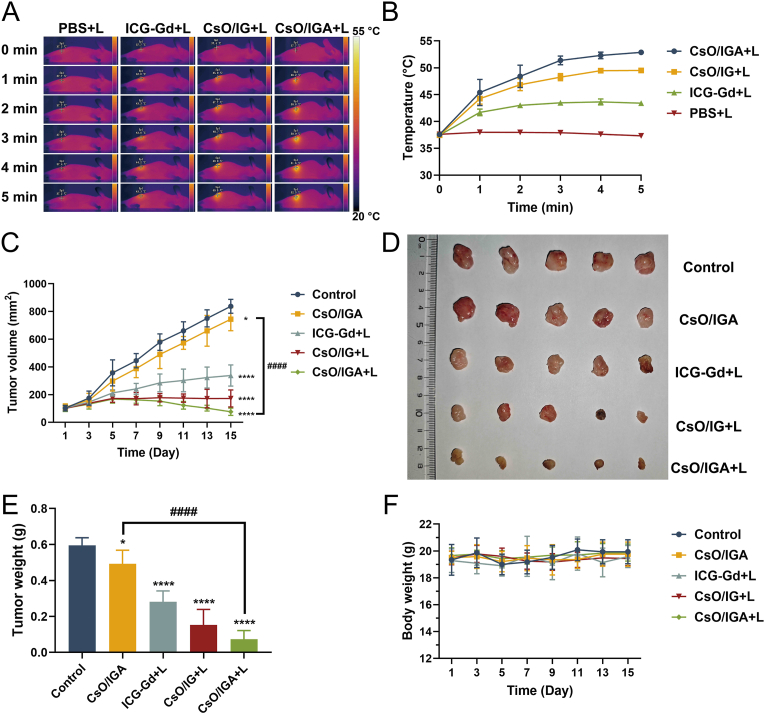
*In vivo* antitumor efficacy in P5 tumor-bearing mice. (A) Representative infrared thermal images and (B) quantitative temperature-time curves at the tumor site of P5 tumor-bearing mice treated with PBS, ICG-Gd, CsO/IG, and CsO/IGA (ICG dose of 1 mg/kg) under 808 nm laser irradiation (1 W/cm^2^, 5 min). (C) Tumor growth curves of P5 tumor-bearing mice over two weeks of treatment. (D) Photographs and (E) weights of excised tumors from different treatment groups after two weeks of treatment. ∗p < 0.05, ∗p < 0.05, ∗∗∗∗p < 0.0001, compared with Control group. ####p < 0.0001 between indicated groups. (F) Body weight changes of P5 tumor-bearing mice monitored throughout the two weeks treatment.

Prior to evaluating the *in vivo* anti-tumor efficacy, we first assessed the photothermal effects in tumor-bearing mice. At 2 h post intravenous injection, the tumor region was subjected to laser irradiation and the temperature changes in the tumor region were monitored using an infrared thermal camera (Fig. 7A and B). The PBS+L group showed negligible temperature changes within 5 min, confirming the minimal thermal effect of the laser. In contrast, ICG-Gd, CsO/IG, and CsO/IGA exhibited rapid temperature increases upon laser irradiation, reaching peak temperatures of 43.6 °C, 49.5 °C, and 52.9 °C, respectively. The moderate hyperthermia achieved by CsO/IGA (52.9 °C) was chosen to achieve effective tumor ablation while avoiding excessive thermal damage to normal tissues [48]. This temperature range expected to synergizes with AZD9291 by enhancing drug release, increasing membrane permeability, and sensitizing resistant cancer cells, thereby overcoming EGFR-TKI resistance.

The *in vivo* therapeutic efficacy were further investigated in subcutaneous P5 tumor-bearing mice model. Control mice receiving PBS injections developed rapidly growing tumors exceeding 800 mm^3^ within 15 days. CsO/IGA treatment showed modest tumor growth suppression, likely due to its antifibrotic properties and enhanced tumor penetration. The laser combination therapy groups demonstrated significantly enhanced tumor suppression through combined PDT/PTT effects. Particularly, the CsO/IGA+L group exhibited initial tumor growth retardation followed by substantial volume reduction in later treatment phases, ultimately achieving the most pronounced anti-tumor effect (Fig. 7C). This therapeutic progression conclusively validates the synergistic action of combined phototherapy and chemotherapy in simultaneously inhibiting tumor growth and overcoming drug resistance. After treatment, the tumor was photographed and weighed (Fig. 7D and E). The CsO/IGA+L group exhibited the smallest tumor volumes and lowest tumor weights among all groups. Furthermore, immunofluorescence staining of α-SMA expression on tumor tissue sections revealed a marked reduction in CAF activation (Fig. S21), demonstrating the TME remodeling capability of OA within the CsO formulation.

During the treatment period, the body weight of P5 tumor-bearing mice were monitored. All nude mice maintained stable body weight throughout the therapy, with no observable signs of anorexia or emaciation (Fig. 7F). The H&E staining revealed no significant abnormalities or pathological changes in major organs (heart, liver, spleen, lung, and kidney) across all treatment groups (Fig. S22). To address potential Gd^3+^ toxicity concerns, we systematically evaluated the biosafety profile of our nanoplatform. ICP-OES analysis revealed that Gd^3+^ residues in the heart, liver, spleen, lung, and kidney were all below the detection limit, indicating negligible accumulation in major organs post-administration. The absence of detectable Gd^3+^ accumulation is likely attributable to the exceptionally low administered dose, which is substantially lower than the standard clinical diagnostic dose of Gd^3+^-based contrast agents. To further systematically evaluate the *in vivo* hepatic and renal biosafety of CsO/IGA nanoparticles, serum levels of ALT, AST, BUN, and CRE were quantitatively determined after administration. As presented in Fig. S23, no obvious changes in ALT, BUN, and CRE were observed between the control and CsO/IGA groups, with no statistical significance. Although a moderate statistically significant increase in AST was detected, the unchanged ALT level excluded severe hepatocellular injury. This isolated AST elevation may reflect temporary metabolic changes rather than true hepatocellular injury, as ALT is a more specific marker of liver damage. Combined with the results of hemolysis assay and H&E pathological staining of main organs, these serum biochemical data collectively verified the negligible acute hepatic and renal toxicity of CsO/IGA *in vivo*. These findings collectively demonstrate the favorable biosafety profile of all tested formulations.

## 4 Conclusions

In this work, we developed a multifunctional nanotheranostic platform CsO/IGA using OA-modified Cs to co-deliver AZD9291 and an ICG-Gd probe, which effectively overcame AZD9291 resistance while integrating chemo-phototherapy with FLI/MRI dual-modal imaging. CsO/IGA exhibited uniform size distribution, excellent colloidal stability, and pH/NIR-responsive drug release properties. Under 808 nm laser irradiation, CsO/IGA effectively generated both ^1^O_2_ and hyperthermia, with Gd^3+^ chelation and CsO encapsulation significantly enhancing the photothermal stability of ICG. CsO/IGA under laser irradiaiton exerted potent antitumor effects against both sensitive and resistant NSCLC cells through ROS generation, MMP reduction, necrosis induction, and EGFR/pEGFR downregulation. Furthermore, CsO/IGA remodeled the TME by inhibiting CAF proliferation and reducing α-SMA expression, thereby enhancing tumor penetration. *In vivo* FLI/MRI studies confirmed significant tumor accumulation and deep tissue penetration of CsO/IGA, while the chemo-phototherapeutic combination achieved superior tumor suppression and drug resistance reversal. This multifunctional system combining targeted chemotherapy, dual-modal phototherapy, TME modulation, and dual-modal imaging capabilities, demonstrates strong potential for clinical translation in overcoming resistance in NSCLC treatment.

## CRediT authorship contribution statement

**Fangying Zheng:** Conceptualization, Data curation, Formal analysis, Investigation, Methodology, Visualization, Writing – original draft. **Yanyun Su:** Data curation, Formal analysis, Investigation, Visualization, Writing – original draft. **Xianbin Sun:** Investigation, Visualization. **Ding Tan:** Investigation, Visualization. **Ya Wang:** Investigation. **Xiumei Li:** Funding acquisition, Project administration, Supervision. **Yu Gao:** Conceptualization, Funding acquisition, Methodology, Project administration, Supervision, Writing – review & editing.

## Declaration of competing interest

The authors declare that they have no known competing financial interests or personal relationships that could have appeared to influence the work reported in this paper.

## Data Availability

The data that support the findings of this study are available from the corresponding author upon reasonable request.
