# Supplementary material for: Chitosan-based nanotheranostics integrated dual-modal imaging and combinatorial tumor therapy for EGFR-TKI resistance reversal
Source: Mater Today Bio. 2026 Jun 29;39:103420. doi: 10.1016/j.mtbio.2026.103420 (PMC13351838; doi:10.1016/j.mtbio.2026.103420)
Supplement: Multimedia component 1 [file mmc1.docx]

Supporting Information

**Chitosan-based nanotheranostics integrated dual-modal imaging and combinatorial tumor therapy for EGFR-TKI resistance reversal**

Fangying Zheng^1,#^, Yanyun Su^1,#^, Xianbin Sun^1^, Ding Tan^1^, Ya Wang^1^, Xiumei Li^2,^*, Yu Gao^1,^*

^1^ Fujian Provincial Key Laboratory of Cancer Metastasis Chemoprevention and Chemotherapy, College of Chemistry, Fuzhou University, Fuzhou, Fujian 350116, China

^2^ Department of Radiology, The First Affiliated Hospital of Fujian Medical University, Fuzhou, Fujian 350005, China

^#^F. Zheng and Y. Su contributed equally to this work.

Corresponding authors:

*Yu Gao (hellogaoyu@126.com or ygao@fzu.edu.cn)

*Xiumei Li (meimei200110011@163.com)


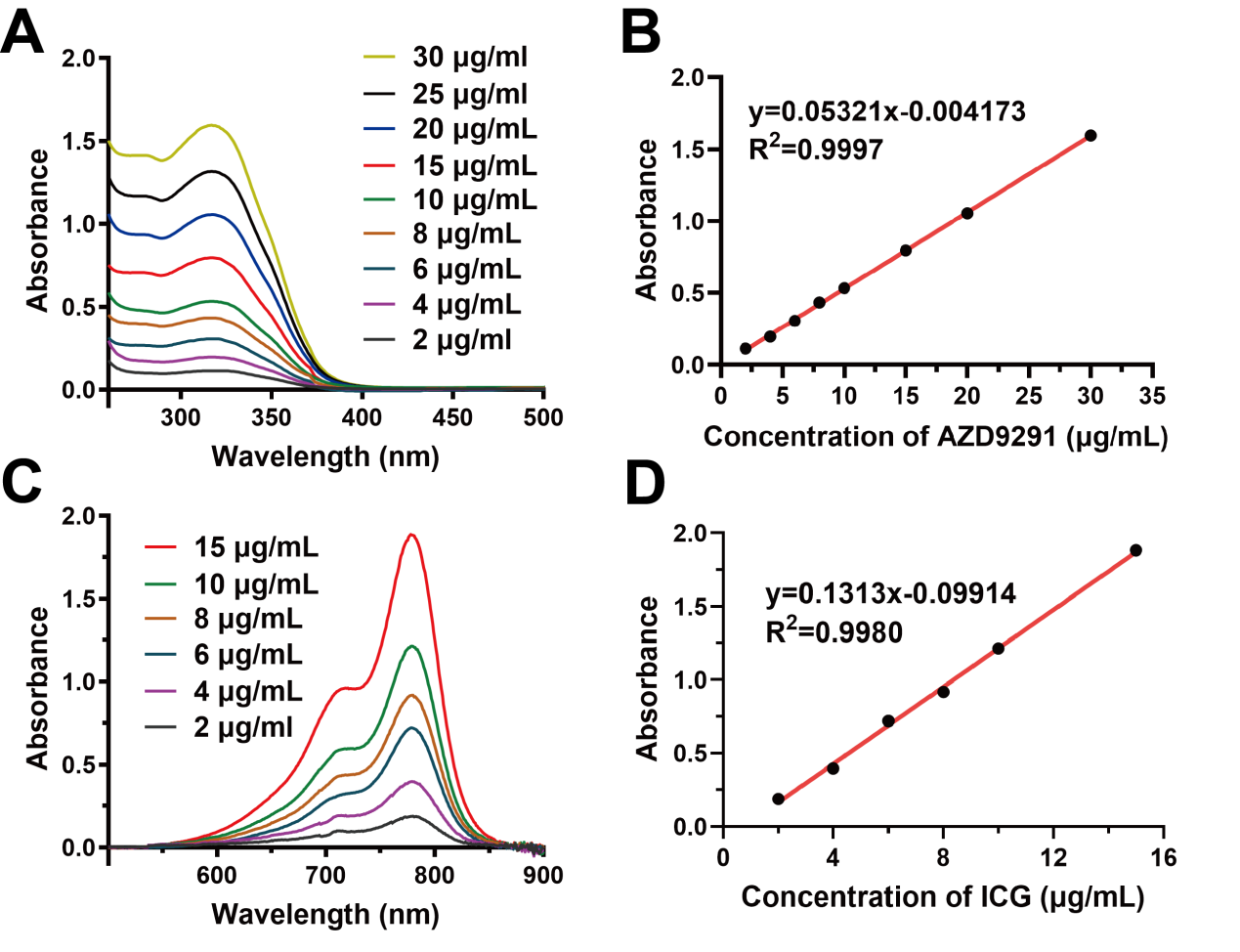


**Fig. S1.** (A) UV-Vis absorption spectra of AZD9291 at different concentrations. (B) Standard calibration curve of AZD9291 obtained by measuring the absorbance at 317 nm. (C) UV-Vis absorption spectra of ICG at different concentrations. (D) Standard calibration curve of ICG obtained by measuring the absorbance at 780 nm.


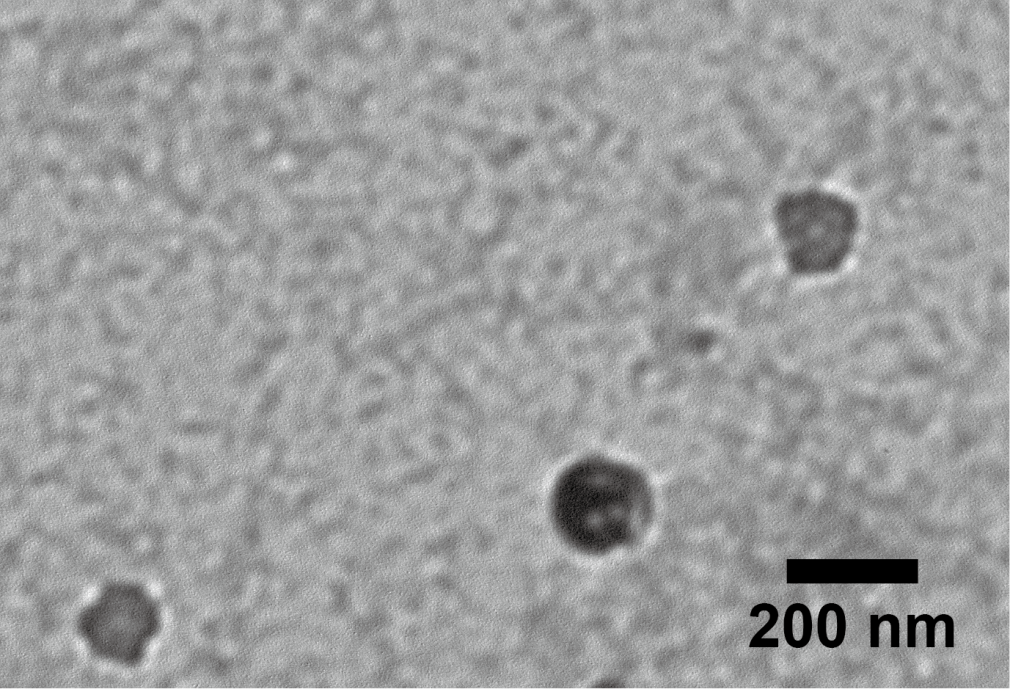


**Fig. S2.** TEM image of CsO.


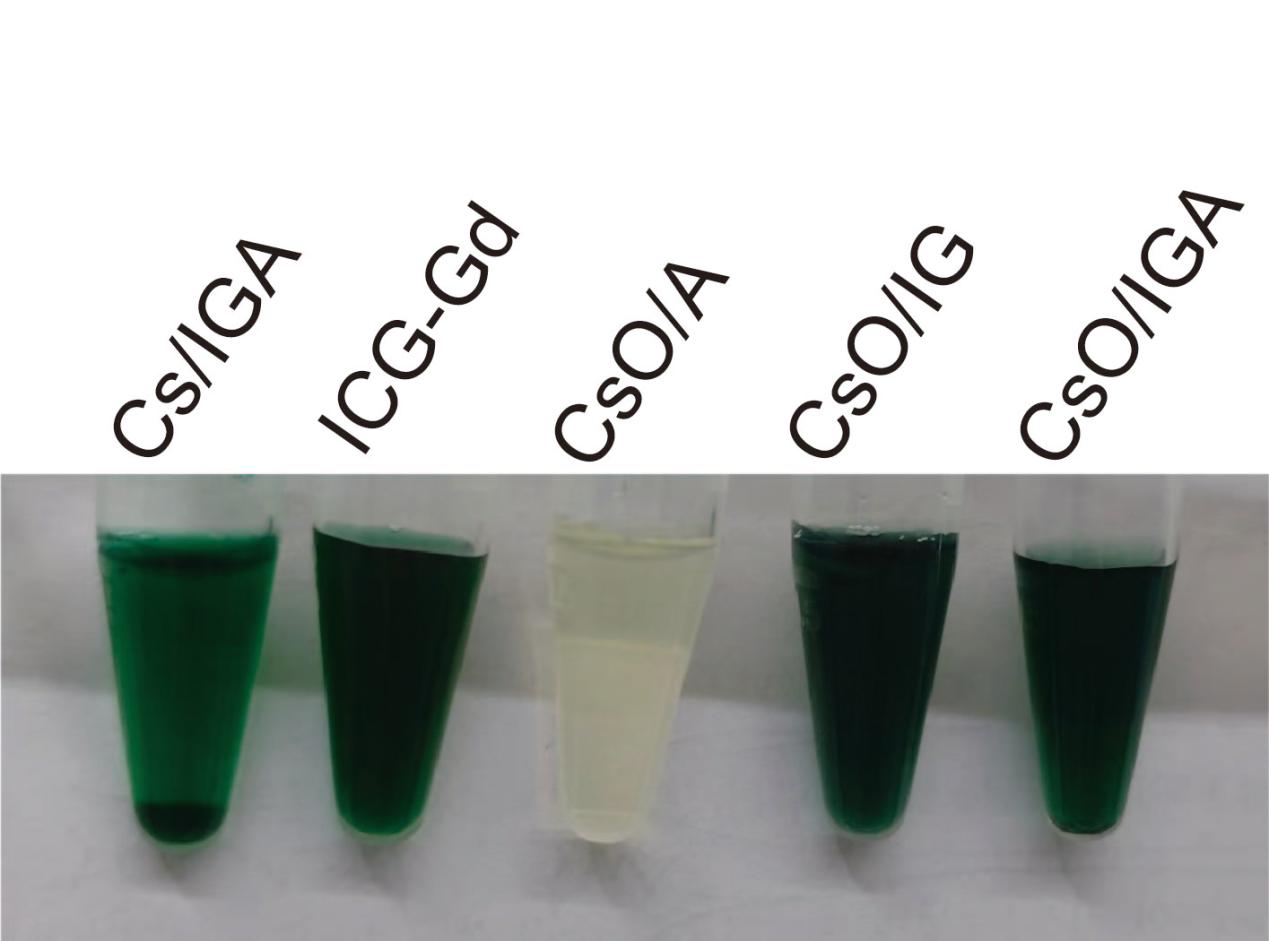


**Fig. S3.** Stability assessment of various formulations in aqueous solution. Photographs of Cs/IGA, ICG-Gd, CsO/A, CsO/IG, and CsO/IGA after standing for 4 h. Cs/IGA immediately formed precipitates, indicating poor stability that precluded its use in further biological evaluations.


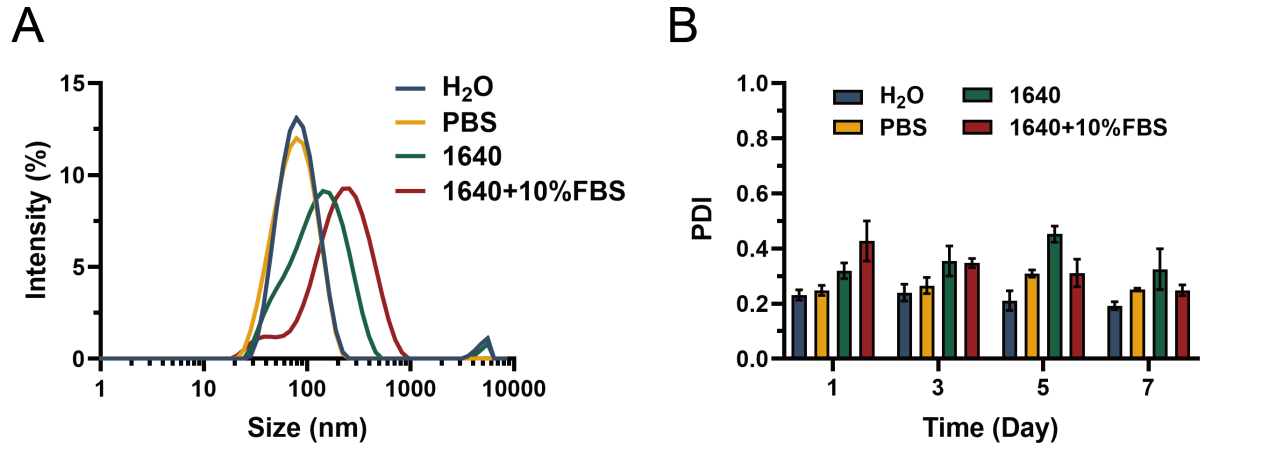


**Fig. S4.** (A) Particle size distribution of CsO/IGA in different media (ddH_2_O, PBS, RPMI-1640, and RPMI-1640+10% FBS). (B) Time-dependent changes in PDI of CsO/IGA in different media over 1 week.

**
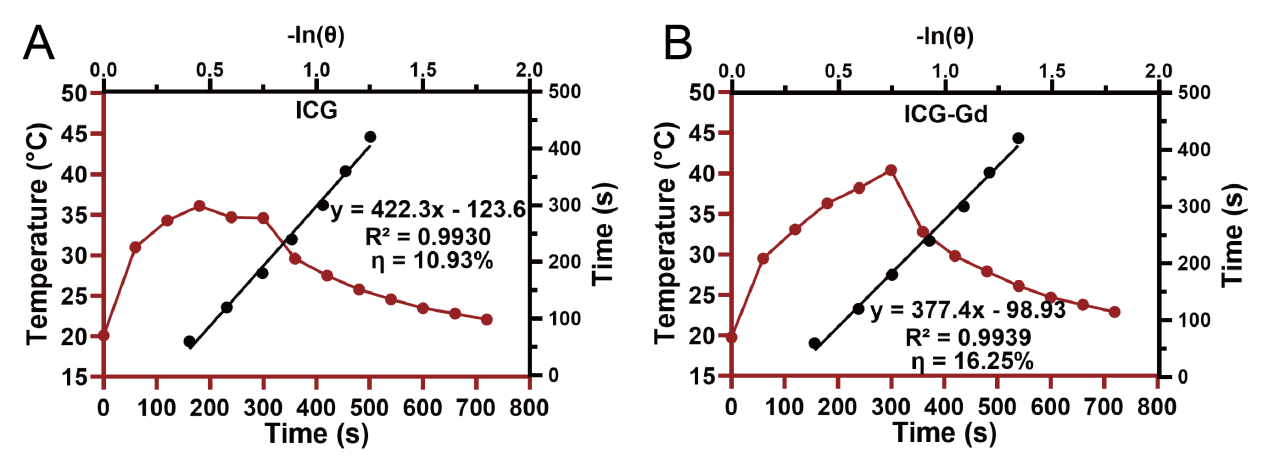
**

**Fig. S5.** Temperature-time curves (red line) and cooling-derived time constants (black line) for PCE calculation of ICG (A) and ICG-Gd (B). Analyses were performed using the first cooling cycle from **Fig. 2F**.


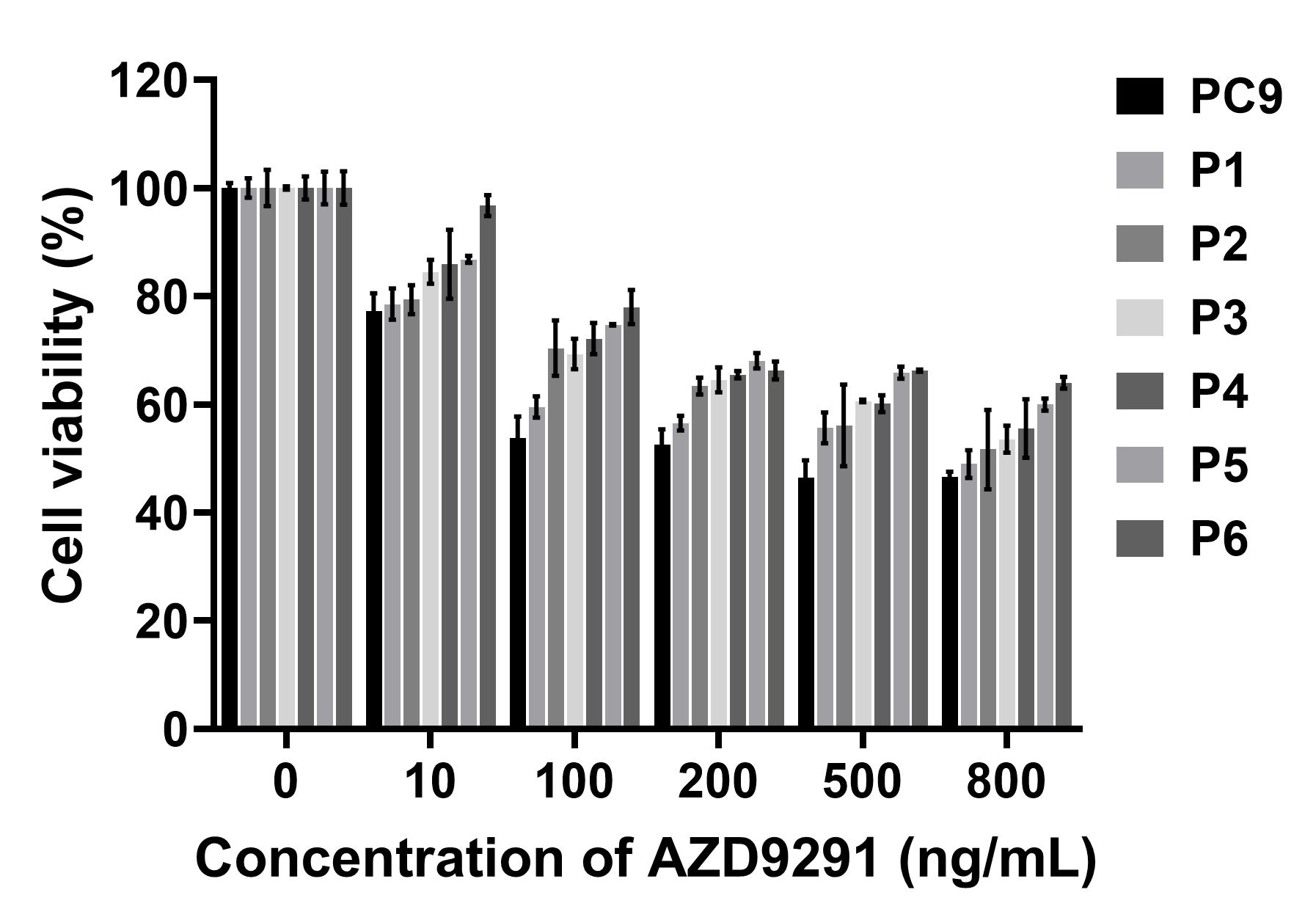


**Fig. S6.** Cytotoxicity assessment of AZD9291 in progressively resistant cell lines (P1-P6) by MTT assay.


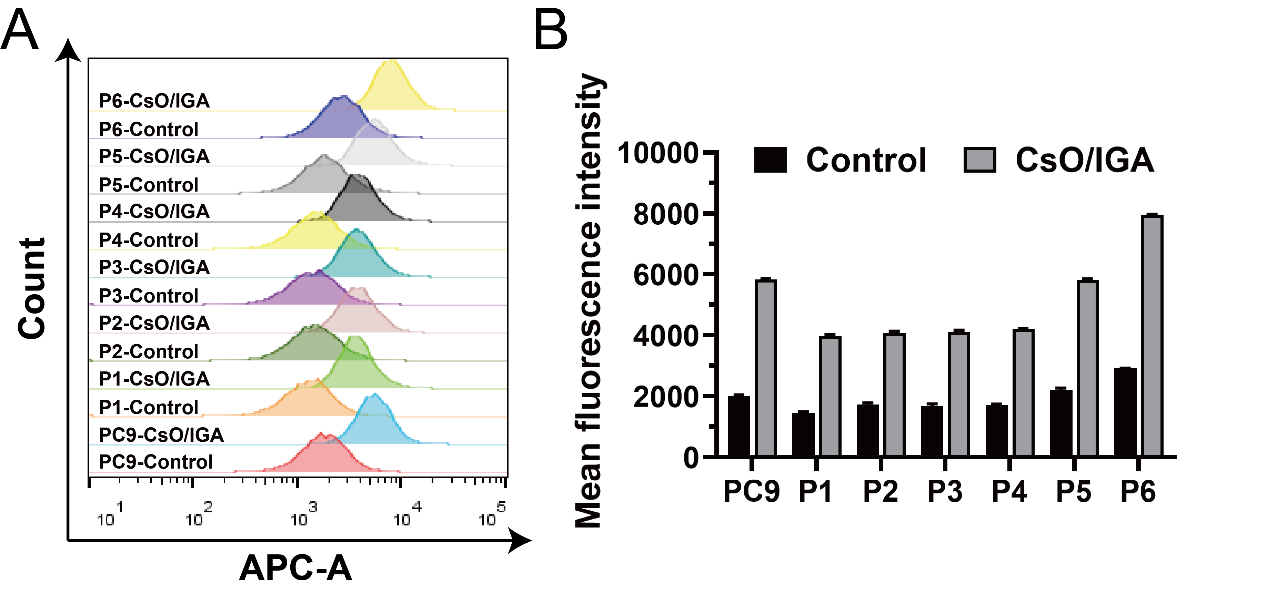


**Fig. S7.** (A) Flow cytometry histograms and (B) quantitative analysis of CsO/IGA uptake in P1-P6 cells after 4 h incubation.


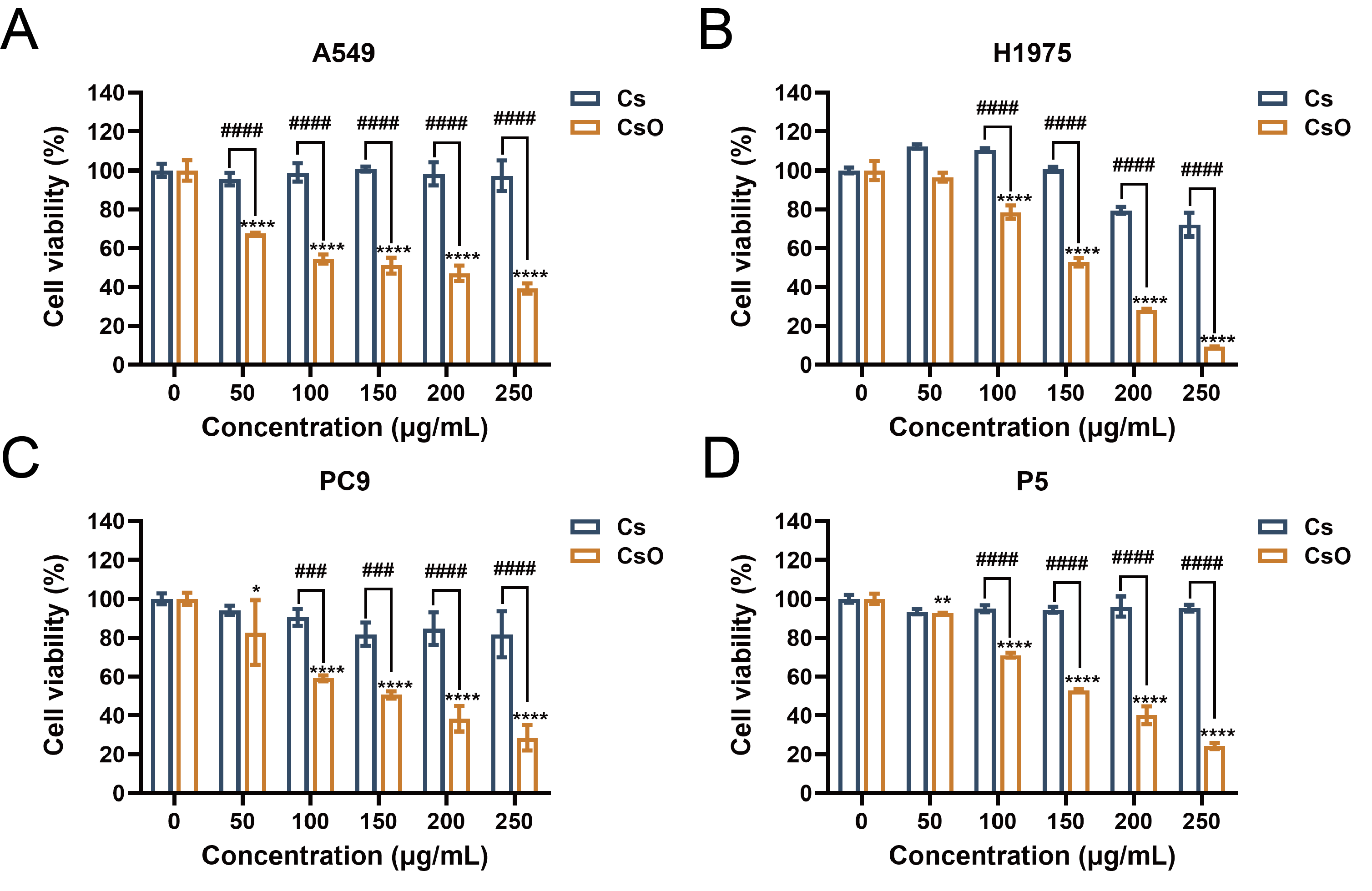


**Fig. S8.** Cell viabilities of A549 (A), H1975 (B), PC9 (C), and P5 (D) cells after treated with Cs and CsO for 48 h. *p<0.05, **p<0.01, ****p<0.0001, compared with Control group. ###p<0.001, ####p<0.0001 for CsO versus Cs treatment.


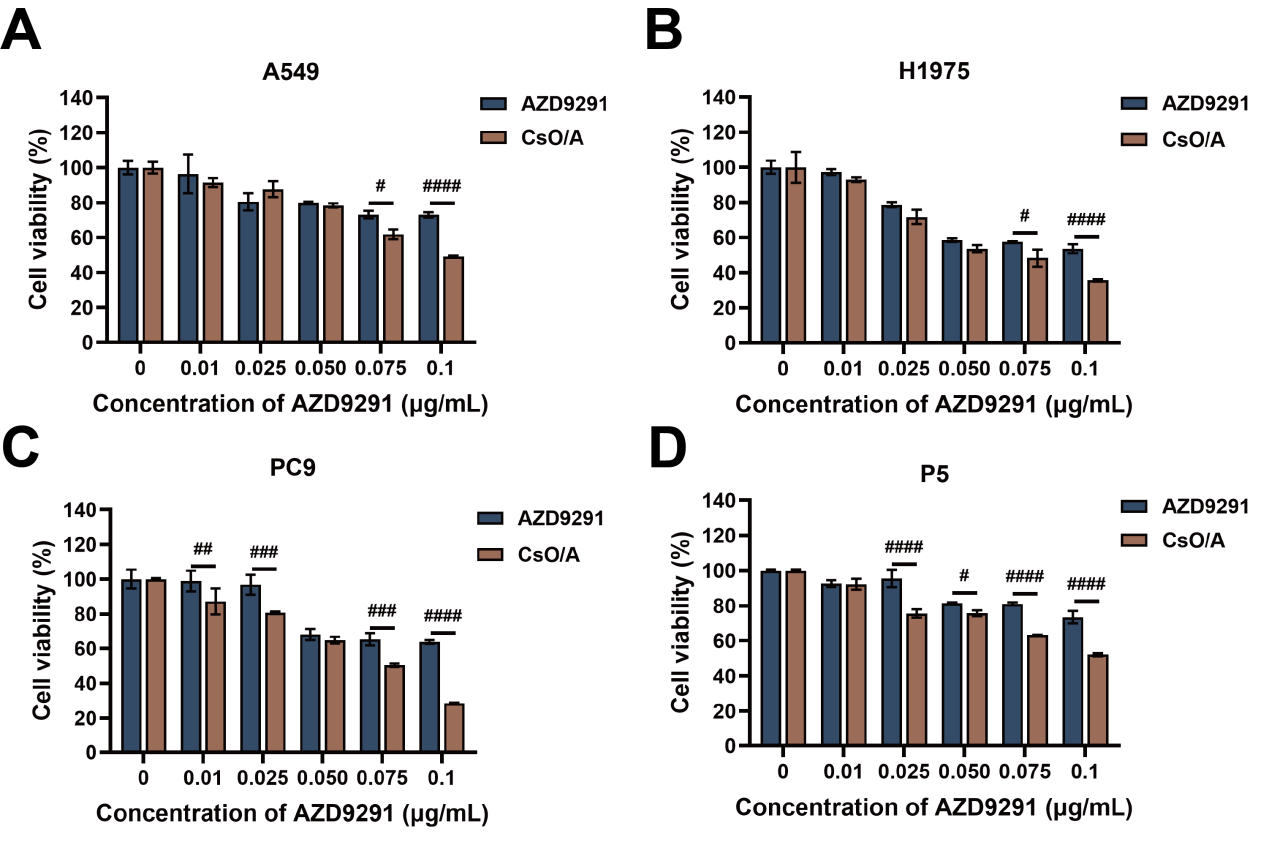


**Fig. S9.** Cell viabilities of A549 (A), H1975 (B), PC9 (C), and P5 (D) cells after treated with free drug AZD9291 and CsO/A for 24 h. #p<0.05, ##p<0.01, ###p<0.001, ####p<0.0001 for CsO/A versus free AZD9291.


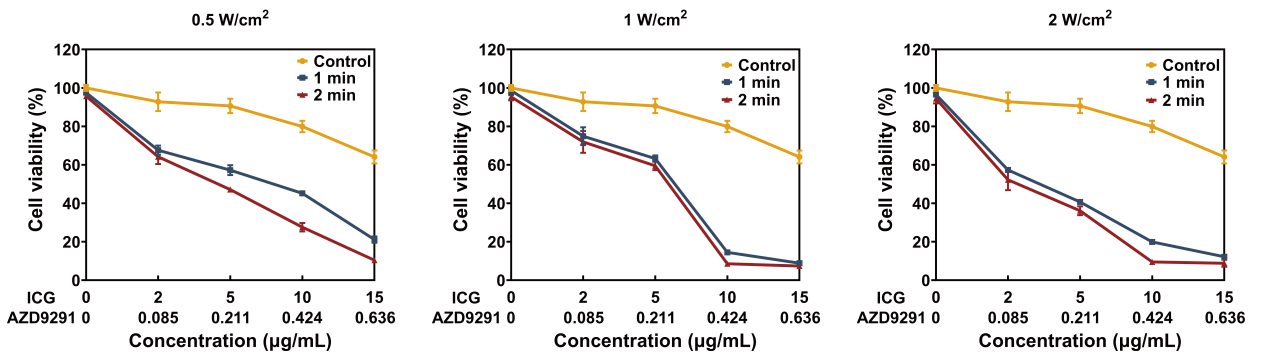


**Fig. S10.** *In vitro* cytotoxicity of CsO/IGA against PC9 cells under different laser irradiation conditions assessed by MTT assay.


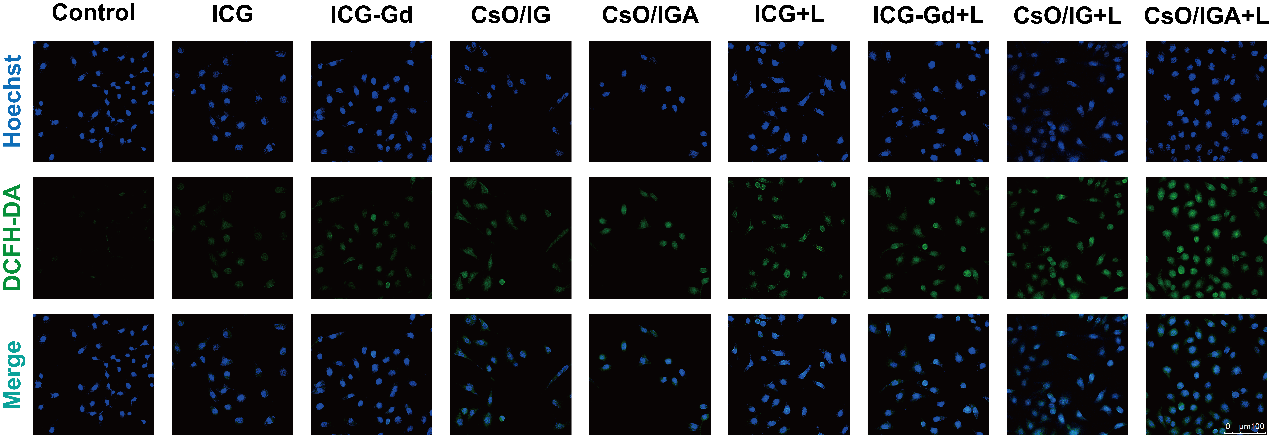


**Fig. S11.** CLSM images showing ROS production in A549 cells following different treatments.


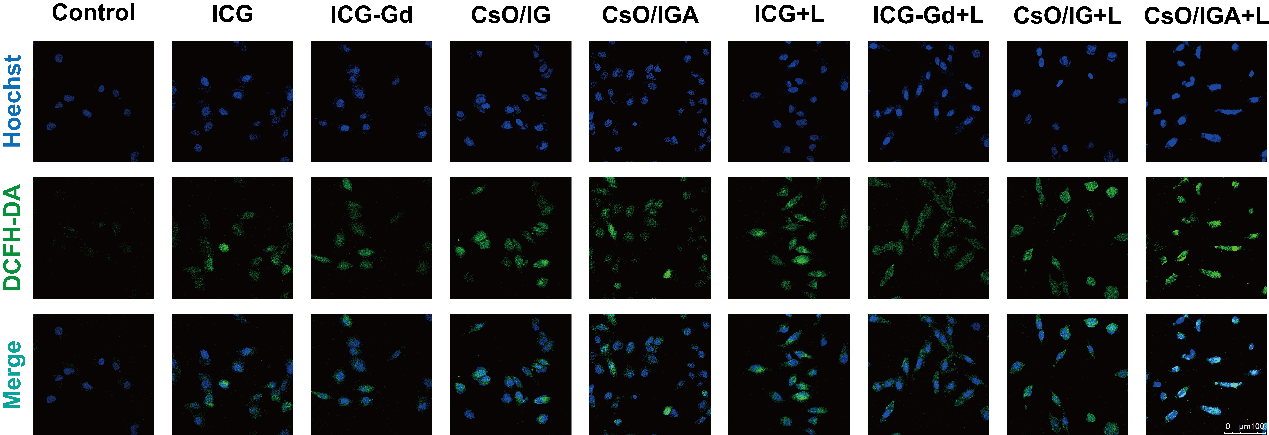


**Fig. S12.** CLSM images showing ROS production in H1975 cells following different treatments.

**
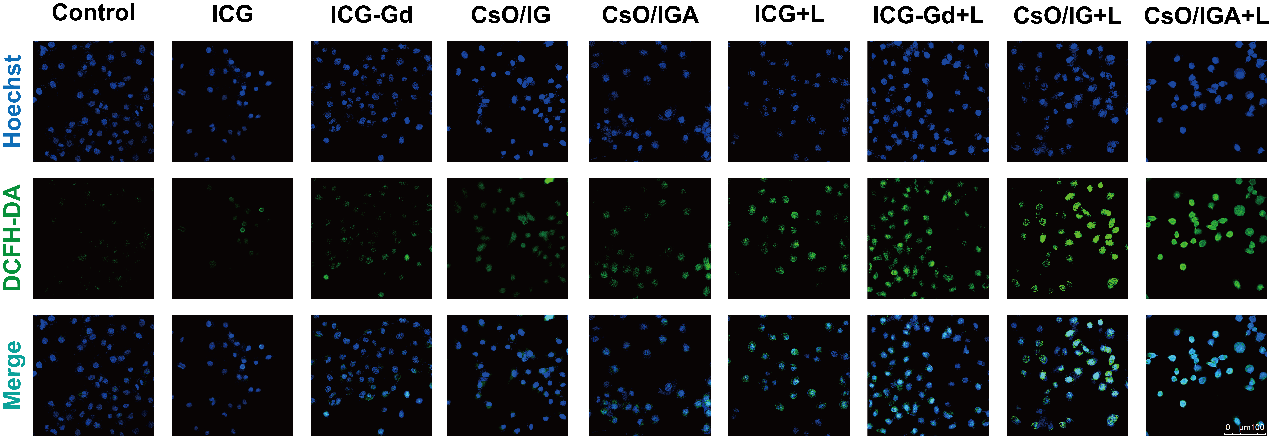
**

**Fig. S13.** CLSM images showing ROS production in PC9 cells following various treatments.

**
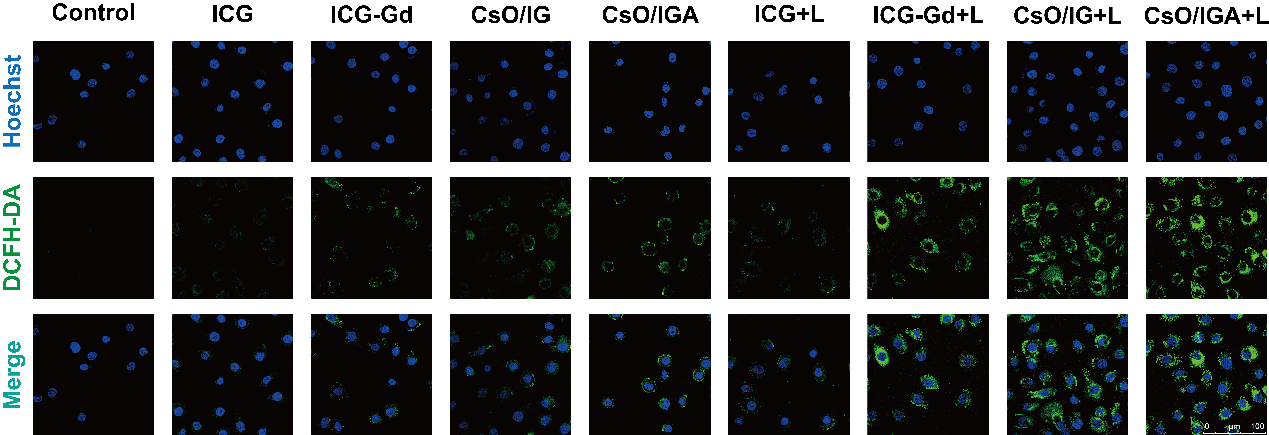
**

**Fig. S14.** CLSM images showing ROS production in P5 cells following various treatments.


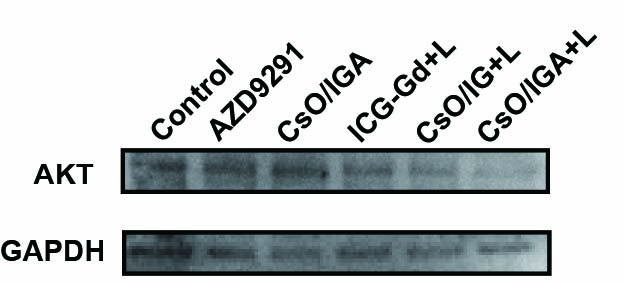


**Fig. S15.** Western blot analysis of total AKT expressions in P5 cells after different treatments.


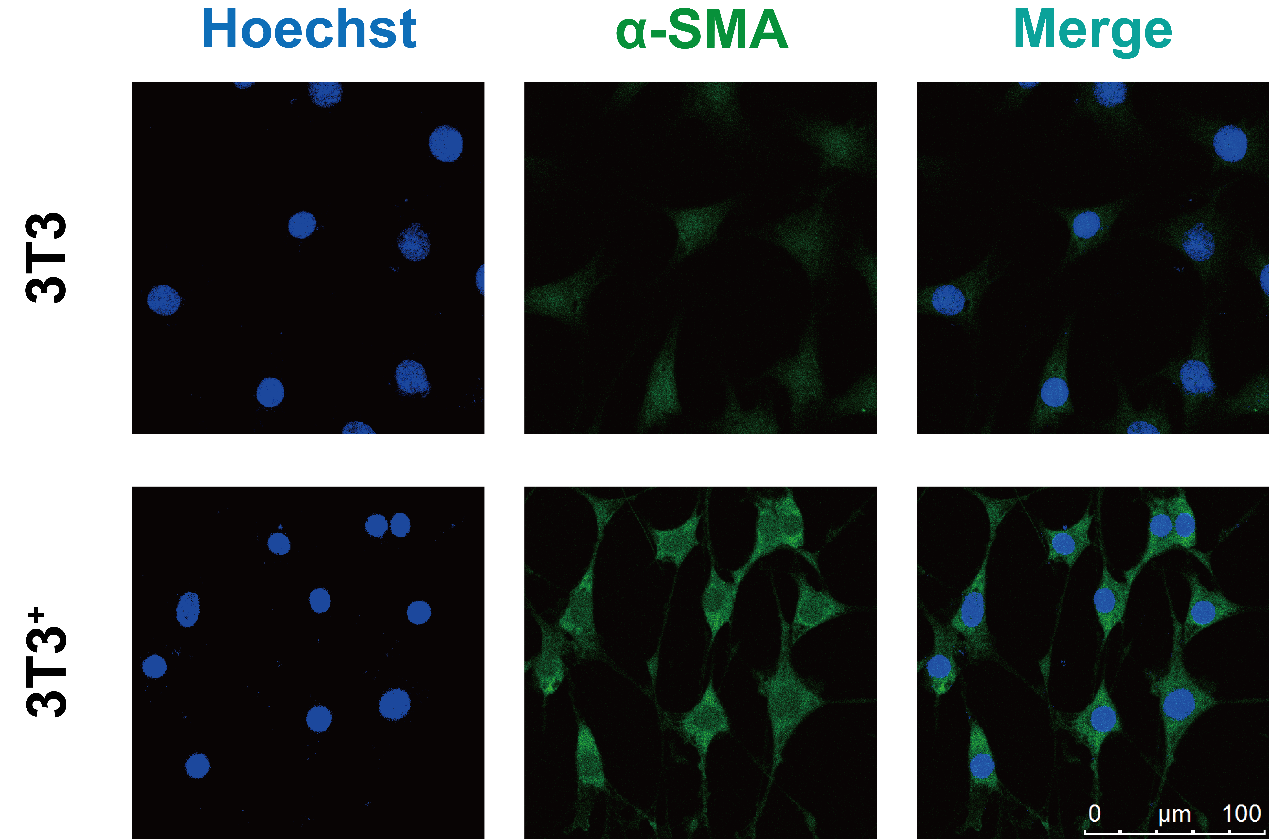


**Fig. S16.** Immunofluorescence staining of α-SMA in 3T3 and 3T3^+^ cells.


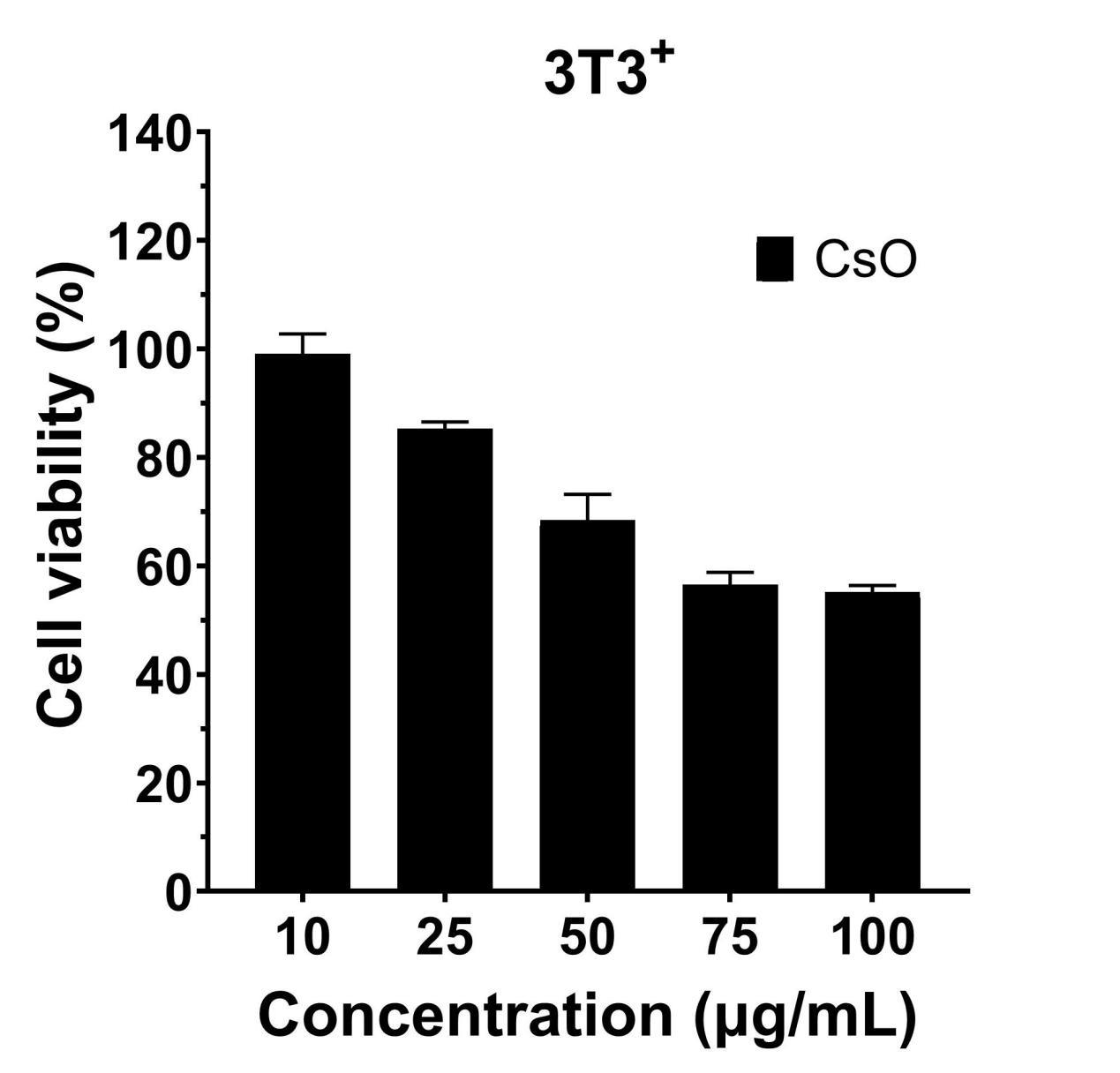


**Fig. S17.** Viability of 3T3^+^ cells after treatment with CsO for 24 h.


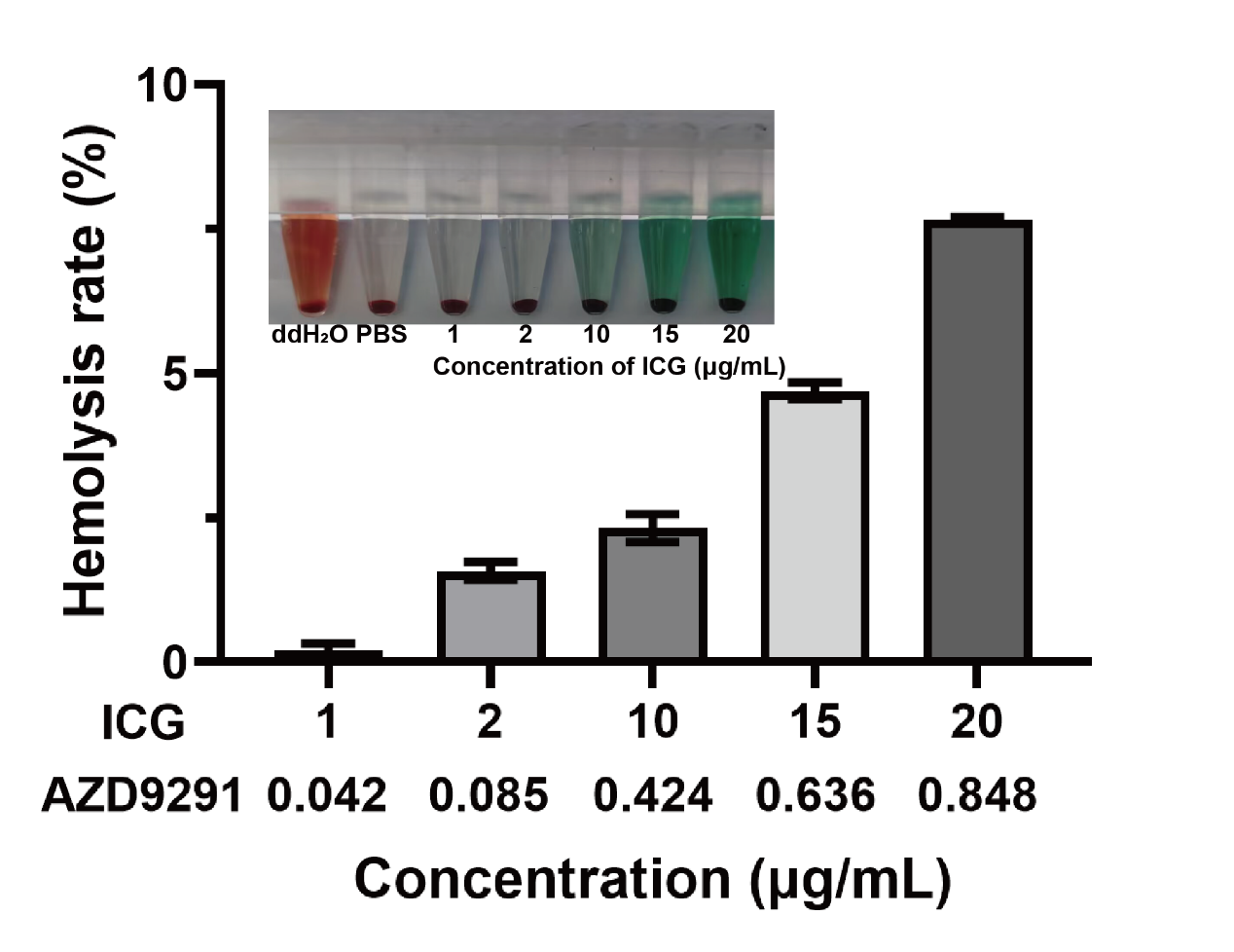


**Fig. S18.** Hemolysis activity of CsO/IGA with varying ICG concentrations (1, 2, 10, 15, 20 μg/mL) in mouse erythrocytes after 3 h incubation.


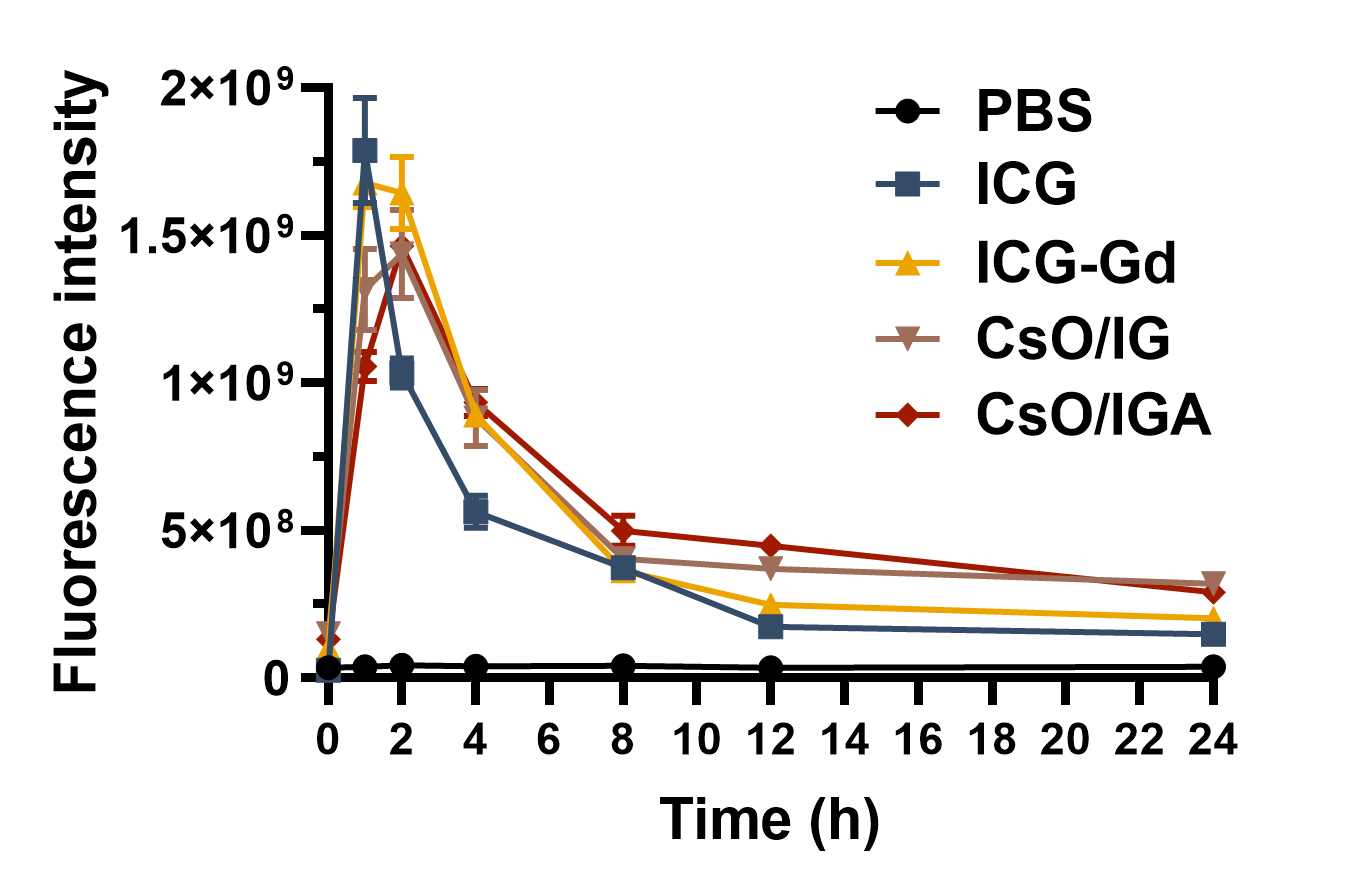


**Fig. S19.** Quantitative analysis of ICG fluorescence intensity at tumor sites.


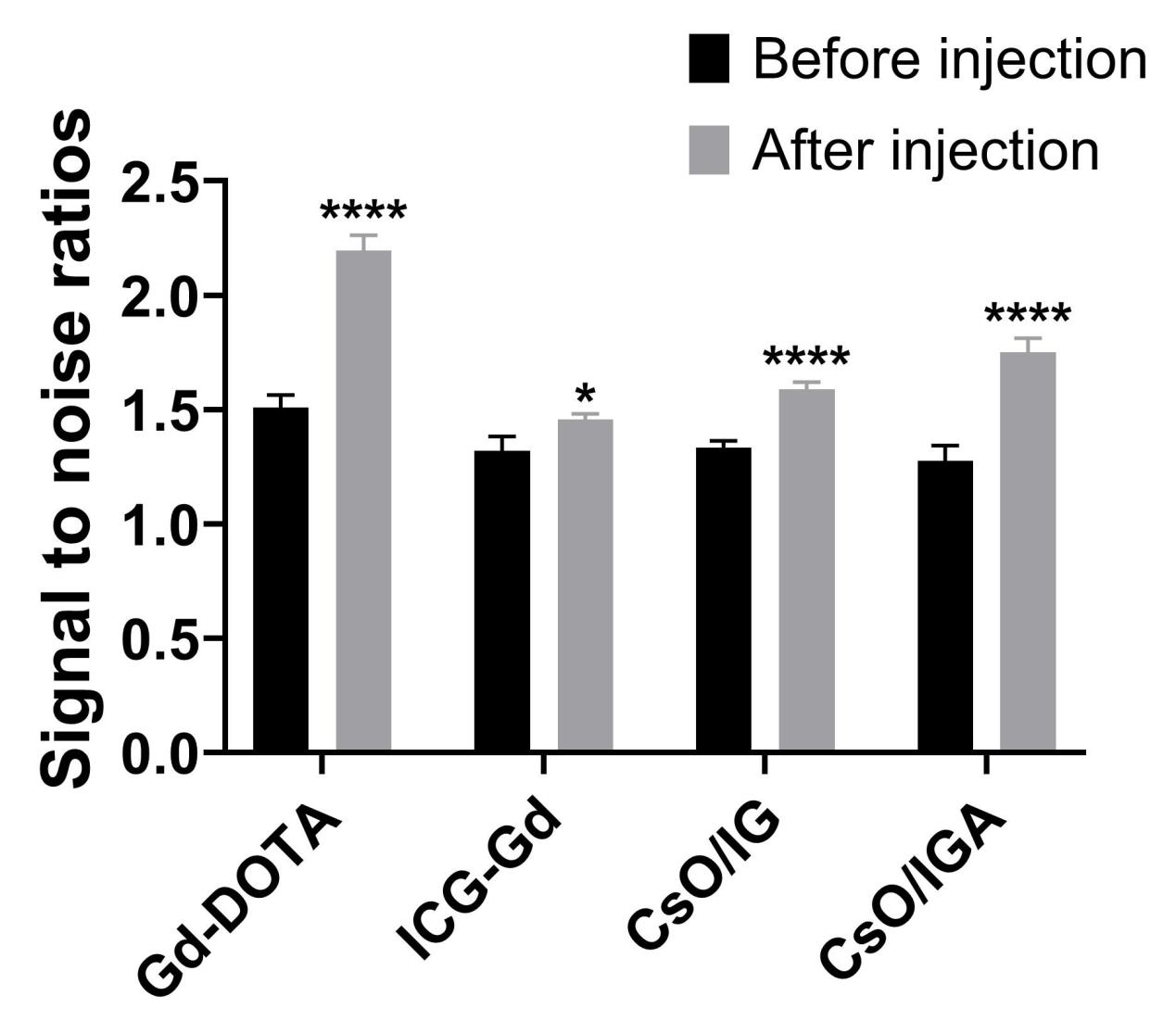


**Fig. S20.** Quantitative analysis of MRI signal-to-noise ratios (SNR) in tumor regions before and after intravenous injection of Gd-DOTA, ICG-Gd, CsO/IG, and CsO/IGA. *p<0.05, **p<0.01, ***p<0.001, ****p<0.0001 compared with before injection.


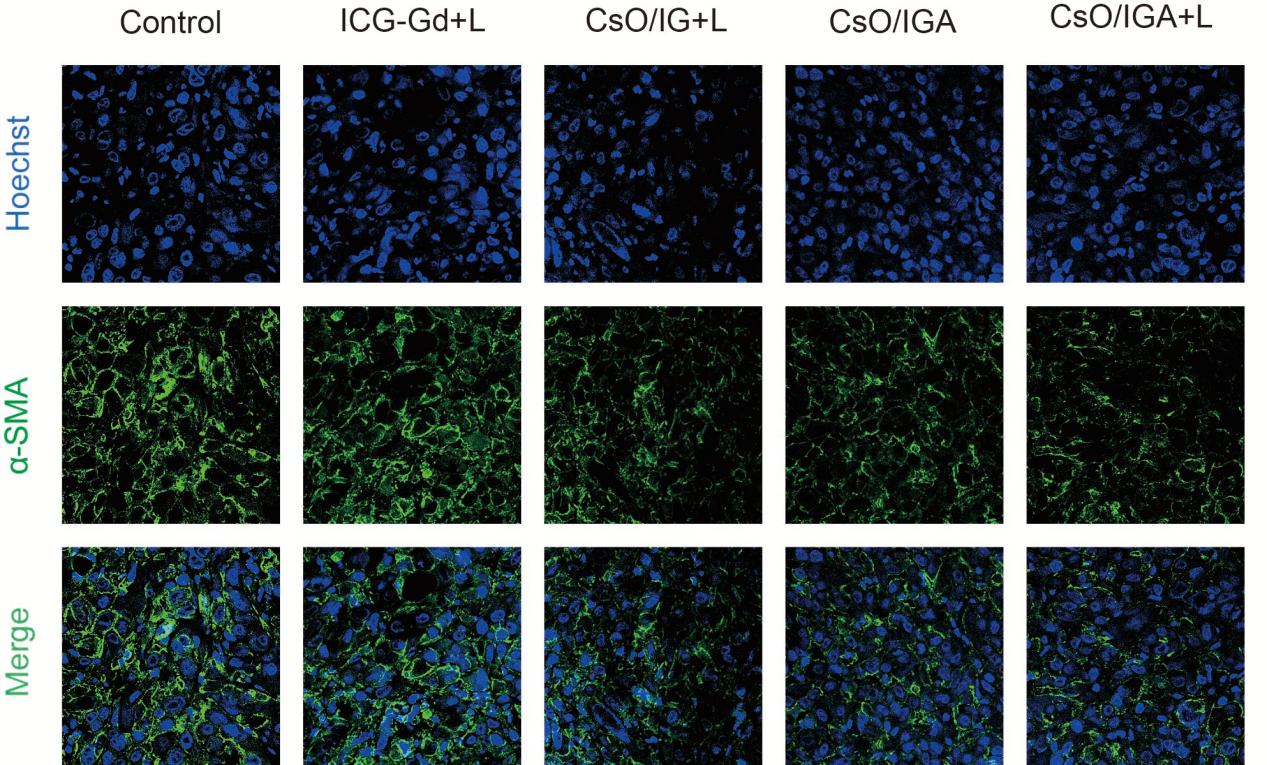


**Fig. S21.** Immunofluorescence staining of α-SMA expression in tumor tissue sections after different treatments.


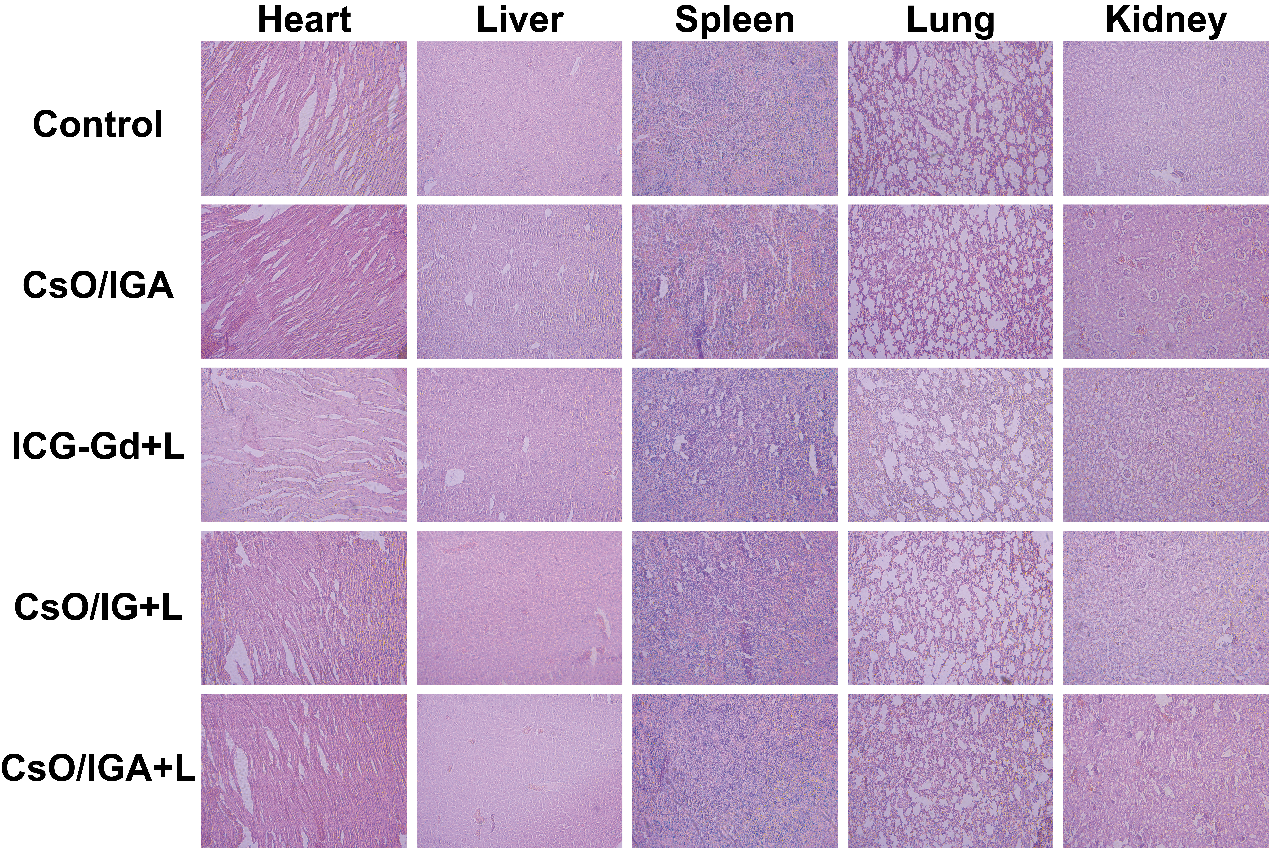


**Fig. S22.** H&E-stained sections of major organs obtained from P5 tumor-bearing mice following different treatments.


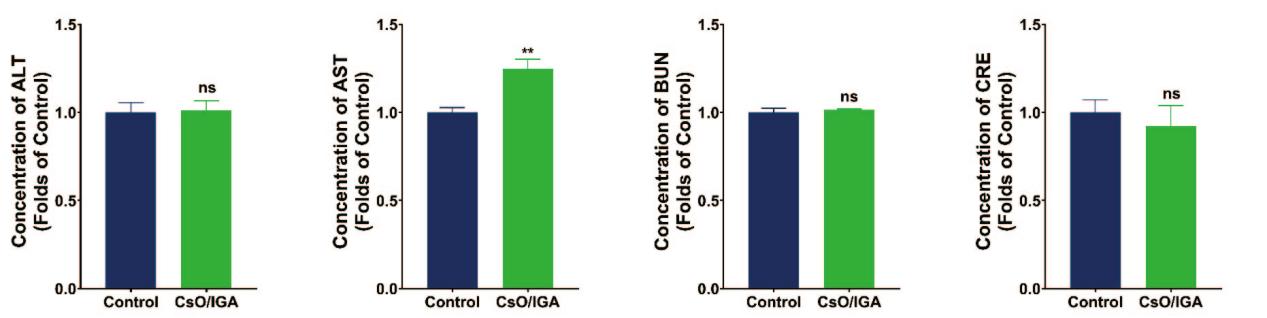


**Fig. S23.** Evaluation of liver and kidney function based on serum biochemical indicators following CsO/IGA treatment. ns: not significant; **p < 0.01 compared with Control group.

**Table S1.** Physicochemical characterization of CsO/A prepared with different weight ratios of CsO to AZD9291.

| Sample | Particle size (nm) | PDI | Zeta potential (mV) | EE of AZD9291 (%) |
| --- | --- | --- | --- | --- |
| CsO NPs | 159.27±6.55 | 0.317±0.031 | -16.1±1.0 | - |
| CsO/A (200:1) | 273.87±4.29 | 0.268±0.023 | −5.59±0.90 | 74.43±2.89 |
| CsO/A (200:2) | 179.53±0.12 | 0.164±0.049 | -9.06±0.05 | 59.35±5.10 |
| CsO/A (200:4) | 230.03±1.86 | 0.229±0.012 | -7.71±1.18 | 53.69±0.76 |
| CsO/A (200:5) | 199.03±1.96 | 0.293±0.017 | -8.22±0.66 | 52.65±0.36 |

**Table S2.** Physicochemical characterization of CsO/IG prepared with different weight ratios of CsO to ICG.

| Sample | Particle size (nm) | PDI | Zeta potential (mV) | EE of ICG (%) |
| --- | --- | --- | --- | --- |
| CsO/IG (200:20) | 108.07±1.68 | 0.412±0.013 | -15.4±0.7 | 56.16±0.69 |
| CsO/IG (200:40) | 81.97±0.50 | 0.167±0.018 | -18.9±0.4 | 62.04±2.12 |
| CsO/IG (200:50) | 111.40±0.16 | 0.141±0.035 | -22.2±0.6 | 63.79±2.54 |

**Table S3.** Physicochemical characterization of CsO/IGA prepared at different CsO:ICG:AZD9291 weight ratios.

| CsO/ICG/AZD9291 | Particle size (nm) | PDI | Zeta potential (mV) | EE of ICG (%) | EE of AZD9291 (%) |
| --- | --- | --- | --- | --- | --- |
| 200:10:2 | 135.40±2.72 | 0.261±0.048 | -12.8±0.9 | 79.91±3.29 | 40.80±4.69 |
| 200:20:2 | 167.37±4.62 | 0.463±0.031 | -18.1±2.2 | 61.77±4.57 | 42.33±4.91 |
| 200:40:2 | 103.57±0.25 | 0.166±0.023 | -19.5±0.9 | 73.32±4.74 | 62.16±0.81 |
| 200:50:2 | 80.23±0.83 | 0.692±0.104 | -15.2±1.1 | 60.92±4.08 | 68.08±1.98 |
